# Exosome-mediated secretion of LOXL4 promotes hepatocellular carcinoma cell invasion and metastasis

**DOI:** 10.1186/s12943-019-0948-8

**Published:** 2019-01-31

**Authors:** Rongkun Li, Yahui Wang, Xiaoxin Zhang, Mingxuan Feng, Jun Ma, Jun Li, Xiaomei Yang, Fang Fang, Qiang Xia, Zhigang Zhang, Mingyi Shang, Shuheng Jiang

**Affiliations:** 1 Department of Interventional Radiology, Tongren Hospital, School of Medicine, Shanghai Jiao Tong University, 1111 Xianxia Road, Shanghai, 200336 People’s Republic of China; 2 State Key Laboratory of Oncogenes and Related Genes, Shanghai Cancer Institute, Ren Ji Hospital, Shanghai Jiao Tong University School of Medicine, 800 Dongchuan Road, Shanghai, 200240 People’s Republic of China; 3 Department of Liver Surgery, Ren Ji Hospital, School of Medicine, Shanghai Jiao Tong University, 160 Pujian Road, Shanghai, 200127 People’s Republic of China

**Keywords:** Hepatocellular carcinoma, LOXL4, Metastasis, Exosomes

## Abstract

**Background:**

Lysyl oxidase-like 4 (LOXL4) has been found to be dysregulated in several human malignancies, including hepatocellular carcinoma (HCC). However, the role of LOXL4 in HCC progression remains largely unclear. In this study, we investigated the clinical significance and biological involvement of LOXL4 in the progression of HCC.

**Methods:**

LOXL4 expression was measured in HCC tissues and cell lines. Overexpression, shRNA-mediated knockdown, recombinant human LOXL4 (rhLOXL4), and deletion mutants were applied to study the function of LOXL4 in HCC. Exosomes derived from HCC cell lines were assessed for the ability to promote cancer progression in standard assays. The effects of LOXL4 on the FAK/Src pathway were examined by western blotting.

**Results:**

LOXL4 was commonly upregulated in HCC tissues and predicted a poor prognosis. Elevated LOXL4 was associated with tumor differentiation, vascular invasion, and tumor-node-metastasis (TNM) stage. Overexpression of LOXL4 promoted, whereas knockdown of LOXL4 inhibited cell migration and invasion of HCC in vitro, and overexpressed LOXL4 promoted intrahepatic and pulmonary metastases of HCC in vivo. Most interestingly, we found that HCC-derived exosomes transferred LOXL4 between HCC cells, and intracellular but not extracellular LOXL4 promoted cell migration by activating the FAK/Src pathway dependent on its amine oxidase activity through a hydrogen peroxide-mediated mechanism. In addition, HCC-derived exosomes transferred LOXL4 to human umbilical vein endothelial cells (HUVECs) though a paracrine mechanism to promote angiogenesis.

**Conclusions:**

Taken together, our data demonstrate a novel function of LOXL4 in tumor metastasis mediated by exosomes through regulation of the FAK/Src pathway and angiogenesis in HCC.

**Electronic supplementary material:**

The online version of this article (10.1186/s12943-019-0948-8) contains supplementary material, which is available to authorized users.

## Background

Hepatocellular carcinoma (HCC), the main type of liver cancer, is one of the most common cancers and is a leading cause of cancer-related death worldwide [1]. Despite emerging improvements in the diagnosis and treatment of HCC, the prognosis is still poor [2]. Although surgical resection is the first-line treatment for HCC, most HCC patients are diagnosed at advanced stages with regional spread and metastasis, which are not eligible for surgical resection or liver transplantation; furthermore, postoperative recurrence and metastasis are quite common, finally leading to death in almost all patients [3]. Therefore, studying the mechanisms underlying HCC metastasis to provide opportunities to improve clinical outcomes and to develop better therapeutic regimens is urgently needed.

The lysyl oxidase (LOX) family has five members, consisting of LOX and lysyl oxidase-like 1–4 (LOXL1–4). They were originally considered copper-dependent amine oxidase and catalyze peptidyl-lysine oxidation in the cross-linking of collagen and elastin, thereby regulating the tensile strength of tissues [4]. All five LOX family members bear a highly conserved catalytic domain in the C-terminus and a signal peptide in the N-terminus with secretory potential, whereas only LOXL2, LOXL3 and LOXL4 possess four scavenger receptor cysteine-rich (SRCR) domains in the N-terminus, which is believed to be for substrate and ligand interaction [5]. Emerging evidence has demonstrated that, in addition to collagen or elastin cross-linking, the LOX family has diverse critical biological functions, especially its roles in tumor development and metastasis [6–8]. Both positive and negative effects of LOX family members in cancers have been reported. Thus far, few studies on the expression and role of LOXL4 in human malignancies are available. Similarly, previous studies have obtained conflicting results regarding the effects of LOXL4 in cancers [9–15]. For instance, our previous study has showed that LOXL4 promotes gastric cancer progression via activating the FAK/Src pathway [11]; LOXL4 knockdown enhances tumor growth and lung metastasis through collagen-dependent extracellular matrix changes in triple-negative breast cancer [14].

Exosomes are a class of small bilayered membrane vesicles (30–150 nm in diameter) that originate from multivesicular bodies (MVBs) and are released into the extracellular environment by many cell types upon fusion of MVBs with the plasma membrane [16, 17]. Secreted extracellular exosomes have the same topology as a cell and contain a broad array of biologically active materials, including proteins, RNA, and DNA [16]. Exosomes have emerged as a major player in intercellular communication through a variety of mechanisms, specifically in cancer development and progression [18–20]. Tumor-secreted exosomes can be internalized by the same and other cell types though autocrine, paracrine, and endocrine mechanisms to facilitate cancer growth and invasion and to promote metastasis [21–24]. Proteins loaded in these exosomes, which to a certain extent reflect the dysregulated protein profile in cancer cells, can thus be transferred to recipient cells to regulate of tumor behaviors. Numerous studies have proved that exosomes play significant roles in liver physiology and pathology, particularly in HCC.

In the present study, we showed that LOXL4 was highly expressed in HCC and predicted a poor clinical prognosis, and promoted migration, invasion, metastasis of HCC by activating the FAK/Src pathway dependent on its amine oxidase activity through a hydrogen peroxide-mediated mechanism. Intriguingly, we found that HCC-derived exosomes transferred LOXL4 proteins between HCC cells, as well as to human umbilical vein endothelial cells (HUVECs), resulting in tumor invasion and metastasis. This study reveals that LOXL4 functions as an oncogene in HCC and may be a promising therapeutic target for HCC.

## Methods

### Clinical samples

Two sets of HCC samples were used in this study. The first set containing 42 HCC samples was used to analyze *LOXL4* expression at mRNA level. The second set containing 254 HCC samples was used to analyze LOXL4 protein expression and to evaluate the correlation with clinicopathological features. All HCC specimens were obtained from patients who underwent surgical resection of their tumors in the Department of Transplantation and Hepatic Surgery, Ren Ji Hospital, School of Medicine, Shanghai Jiao Tong University, except for 52 cases, which were purchased from Shanghai Outdo Biotech Inc. (OD-CT-DgLiv01–012). Written informed consent was obtained from each patient involved in this study, and all protocols were approved by the ethical review committee of the World Health Organization Collaborating Center for Research in Human Production (authorized by the Shanghai Municipal Government).

### Cell culture

The human HCC cell lines SK-Hep1 and SUN-423 were obtained from the American Type Cell Culture Collection (ATCC), Hep3B and Huh7 were purchased from the Cell Bank of the Chinese Academy of Sciences, and SMMC-7721, MHCC-97 L and MHCC-LM3 were preserved in Shanghai Cancer Institute, Ren Ji Hospital, School of Medicine, Shanghai Jiao Tong University. All HCC cell lines were cultivated in Dulbecco’s modified Eagle’s medium (DMEM, Gibco) supplemented with 10% fetal bovine serum (FBS, Hyclone). HUVECs were purchased from ATCC and cultivated in endothelial cell complete medium containing endothelial cell growth supplement (Allcells, USA). For hypoxic culture, HCC cells were placed in a hypoxia incubator in an atmosphere consisting of 1% O_2_, 5% CO_2_, and 94% N_2_. PP2 was purchased from Selleck (Shanghai, China), GW4869 and catalase from Sigma-Aldrich.

### Quantitative real-time polymerase chain reaction (qRT-PCR)

Total RNA was isolated from HCC tissues and cell lines using TRIzol RNA isolation reagent (Takara, Japan) and reverse transcribed using a PrimeScript qRT-PCR kit (Takara, Japan) according to the manufacturer’s instructions. QRT-PCR was performed with an SYBR(R) PrimeScript RT-PCR Kit (Takara, Japan) using an ABI7500 system (Applied Biosystems, USA) and with specific primers: LOXL4 [forward 5’-CCGCTGCAAGTATGATGG-3′; reverse 5’-GTTCCTGAGACGCTGTTC-3′], 18sRNA [forward 5’-TGCGAGTACTCAACACCAACA-3′; reverse 5’-GCATATCTTCGGCCCACA-3′]. Relative *LOXL4* gene expression analysis was performed using the eq. 2^-ΔCt^ [ΔCt = Ct (LOXL4) – Ct (18sRNA)], with 18sRNA used as an internal control.

### Immunohistochemical (IHC) staining

IHC staining was performed using a two-step protocol as previously described [25]. Briefly, after deparaffinized with xylene, rehydrated in graded ethanol, immersed in 0.3% hydrogen peroxide, and heat-mediated antigen retrieval in citric acid at pH 6.0, tissue section was incubated with the antibody for LOXL4 (Abcam, ab88186) at 4 °C overnight, labeled by HRP (rabbit) second antibody (Thermo Scientific, USA) at room temperature (RT) for 60 min. Finally, sections were developed in DAB solution (Gene Tech, Shanghai, China) under microscopic observation and counterstained with hematoxylin. The scoring of LOXL4 expression was performed according to both of the ratio and intensity of positive-stained cells: 0–5% scored 0; 6–35% scored 1; 36–70% scored 2; and more than 70% scored 3. The final scores were designated as low or high expression as follows: low expression (score 0–1), high expression (score 2–3). These scores were determined independently by two experienced pathologists in a blinded manner, and mean percentage values were taken.

### Western blotting

Total cell protein and exosome protein were obtained using an IP lysis buffer (Beyotime, Shanghai, China) for further assays. The secreted proteins in conditioned media (CM) were collected by ethanol precipitation. Briefly, 95% ethanol was added to the CM and maintained at − 20 °C overnight. The precipitated proteins were collected with SDS loading buffer and immediately underwent standard western blotting. Western blotting was performed using SDS-PAGE gels for protein separation and nitrocellulose membranes (Millipore, USA) for protein blotting. Membranes were blocked with 5% nonfat milk (BD Biosciences, USA) in Tris-buffered saline (TBS) and incubated with primary antibodies diluted in TBS containing 1% bovine serum albumin at 4 °C overnight. Bound antibodies were detected using an Odyssey Imaging System (LI-COR Biosciences, USA) with DyLight fluorescent dye labeled secondary antibodies. The following antibodies were used: LOXL4 (Abcam, ab88186), CD63 (Abcam, ab68418), CD9 (Abcam, ab92726), TSG101 (Abcam, ab30871), GAPDH (Abcam, ab9482), HA (Millipore, 05-902R), p-FAK (Cell Signaling Technology, #3281), FAK (Cell Signaling Technology, #3285), p-Src (Abcam, ab4816) and Src (Abcam, ab16885).

### Immunofluorescence (IF) staining

All the HCC cell lines used in the tests were seeded on cover slides in 24-well plates and incubated overnight. Cells were fixed in 4% paraformaldehyde for 30 min, permeabilized with 2% Triton X-100 for 2 min, blocked in 1% bovine serum albumin for 60 min, and incubated with primary antibodies against LOXL4 (Abcam, ab88186) for 60 min at RT, followed by an Alexa Fluor 594-conjugated secondary antibody (Molecular Probes, USA) for 30 min at RT. Nuclei were counterstained with 4′,6-diamidino-2-phenylindole dihydrochloride (DAPI, Sigma-Aldrich, USA) at RT for 5 min. IF signals were captured using a laser confocal microscopy (Leica Microsystems AG).

### siRNA transfection

All transfections were performed using Lipofectamine 2000 (Invitrogen, Carlsbad, CA, USA) according to the manufacturer’s instructions. Cells were transfected with SMAD4 siRNAs or negative control (GenePharma Inc., Shanghai, China) at a concentration of 50 nM.

### Establishment of stable overexpression or knock-down cell lines

For overexpression of LOXL4 in HCC cell lines, the full-length cDNA encoding LOXL4 (NM_032211) was subcloned into the pCDH-CMV-MCS-EF1-Puro vector (System Biosciences). After virus packaged in 293 T cells using Lipofectamine 2000, LOXL4 or mock vectors were transfected into target cell lines. For knockdown of LOXL4, shRNA sequences targeting LOXL4 (shLOXL4–1: 5’-CCCTAACATGGGCTTTCAGAA-3′; shLOXL4–2: 5’-CAGGAAAGTCTGGGATCTGAA-3′) were cloned into a pLKO.1-TRC cloning vector (Roche). ShRNA-containing plasmids were packaged and transfected into target cell lines as overexpression. Transfected cells used for overexpression or knockdown were selected with puromycin (5 μg/ml) for 2 weeks.

### Generation of deletions in the LOXL4 SRCR and catalytic domains

The cDNAs encoding full-length LOXL4, SRCR domain deletion (ΔSRCR), and catalytic domain deletion (ΔC-T) were amplified by PCR and subcloned into the pCDH-CMV-MCS-EF1-Puro vector. Primer sequences were listed in Additional file 1: Table S1. These constructs were packaged and transfected into target cell lines as described above, and the transfected cells were also selected with puromycin (5 μg/ml) for 2 weeks.

### LOXL4 enzymatic activity assay

LOXL4 enzymatic activity of whole-cell lysates was measured using the Amplex Red Hydrogen Peroxide/Peroxidase Assay Kit (Invitrogen). Protein samples were prepared in buffer in a 96-well plate and incubated with the reaction mixture for 30 min according to the manufacturer’s instructions. Fluorescence was measured with excitation at 560 nm and emission at 590 nm using a fluorescence microplate reader (BMG Labtech).

### Recombinant human LOXL4 (rhLOXL4) expression, purification and characterization

The cDNA encoding LOXL4 with the signal peptide truncated was amplified by PCR and subcloned into the episomal expression vector pCEP-Pu-Strep II-tag (N-terminal) in-frame with the sequence of the BM-40 (SPARC/osteonectin) signal peptide downstream of the CMV promoter. Briefly, the expression vector was transfected into human embryonic kidney 293/Epstein-Barr nuclear antigen cells (EBNA-293) using X-treme GENE 9 DNA Transfection Reagent (Roche). The transfected cells were cultured with 5 μg/ml puromycin initially for screening and were subsequently cultured at a large scale with 1 μg/ml puromycin. The culture media was collected and centrifuged and the supernatants were applied to the StrepTactin sepharose column (IBA). The column was washed with binding buffer and eluted by elution buffer containing 2.5 mM desthiobiotin. The collected fractions were further quantified and identified by western blotting.

### CM preparation and exosome isolation

The indicated HCC cells at 80% confluence were incubated in serum-free DMEM. Three days later, the CM were collected, filtered, and stored at − 80 °C for up to 2 months. Exosomes were isolated from CM by differential centrifugation. Briefly, CM was collected and centrifuged at 300×g for 30 min, 3000×g for 30 min, 20,000×g for 30 min, and 100,000×g for 80 min at 4 °C. The pellets were washed twice with PBS and purified by centrifugation at 100,000×g for 80 min at 4 °C. The purified exosomes were resuspended in PBS for functional assays or used for protein detection. The protein concentration of exosomes was determined using a BCA Protein Assay Kit (Thermo Fisher Scientific). The size and quality of exosomes was determined using a LM10-HS NanoSlight instrument and nanoparticle tracking analysis (NTA) software.

### Transmission electron microscopy

Purified exosomes from HCC cells were resuspended in PBS, dropped onto electron microscopy grids, and allowed to absorb for 10 min. The absorbed exosomes were then negatively stained with 2% phospphotungstic acid (pH 6.8) for 5 min. After air-dried under an electric incandescent lamp, the electron microscopy grids were examined using a transmission electron microscope (PHLIPS-TECNAI 10) at 120 kV.

### Exosome labeling and tracing

Isolated exosomes were labeled with a PKH26 Red Fluorescent Cell Linker Kit (Sigma, St. Louis, MO) according to the manufacturer’s protocol. Labeled exosomes were resuspended in DMEM and added to a subconfluent layer of HCC cells or HUVECs, which were then incubated at 37 °C for 2 h in a CO_2_ incubator. Cells were washed thrice with PBS, fixed with 4% paraformaldehyde for 30 min, and stained with DAPI for 20 min at RT. Finally, cells were observed under a confocal laser scanning microscope (Leica Microsystems AG).

### Cell proliferation assay

Cells were seeded into 96-well plates in triplicate at a density of 1.5 × 10^3^ cells per well. Cell viability was measured at the indicated time points, using the Cell Counting Kit (CCK8, Dojindo, Japan) according to the manufacturer’s instructions. The absorbance at 450 nm was measured using a multifunctional microplate reader (Bio-Rad Laboratories, Hercules, CA).

### Transwell migration and invasion assays

HCC cells were seeded into Transwell chambers (Millipore, USA) in triplicate at a density of 5 × 10^4^ cells per well in 200 μl serum-free DMEM medium. Then 750 μl DMEM containing 10% FBS, rhLOXL4, CM or exosomes was added into the lower compartment of the Transwell chamber. Cells were allowed to migrate for 24 h or invade through the Matrigel (BD Biosciences) for 48 h. Migrated cells were fixed with 4% paraformaldehyde and stained with 0.2% crystal violet, and then counted under a light microscope at a magnification of 200.

### Anoikis assay

For induction of anoikis, 12-well culture plates were completely covered with poly-hydroxyethyl methacrylate (HEMA, Sigma-Aldrich), prepared as a 10 mg/ml solution in ethanol, then died. Cells in serum-free medium were seeded into the coated plates. At the designated time points, the suspended cells were subjected to Annexin V/PI staining (BD Pharmingen, San Diego, CA) according to the manufacturers’ instruction and analyzed by flow cytometry on a FACS Calibur (Becton Dickinson, San Diego, CA).

### Cell adhesion assay

HCC cells were trypsinized, counted and then plated at a density of 3 × 10^4^ cells per well in 96-well plates coated with collagen I, collagen IV, laminin or fibronectin (10 μg/ml). The cells were incubated at 37 °C for 1 h. The attached cells were fixed with 1% glutaraldehyde for 30 min and stained with 1% crystal violet in water for 30 min. After washing with distilled water, the stained cells were extracted with 0.1% Triton X-100. The absorbance of each well of the plates was measured at 570 nm.

### In vitro vascular tube formation assay

The assay was performed with an In Vitro Angiogenesis Assay Kit (Millipore, Billerica, MA, USA) according to the manufacturer’s instruction. 1 × 10^4^ HUVECs per well were seeded into a 96-well tissue culture plate. HUVECs were treated with exosomes derived from HCC cells, and tube formation was inspected after incubating for 6 h at 37 °C.

### In vivo metastasis assays

Approximately 2 × 10^6^ SK-Hep1 cells stably transfected with LOXL4 or vector were suspended in 40 μl serum-free DMEM/Matrigel (1:1) for each nude mouse. Through a 1 cm transverse incision in the upper abdomen under anesthesia, each nude mouse (6 in each group, 6-week-old male BALB/c-nu/nu) was orthotopically inoculated in the left hepatic lobe using a microsyringe. Another 2 × 10^6^ cells were injected intravenously into nude mice (6 in each group, 6-week-old male BALB/c-nu/nu). After 6 weeks, the mice were sacrificed, and their livers and lungs were dissected, fixed with phosphate-buffered neutral formalin and prepared for standard histological examination. Mice were manipulated and housed according to protocols approved by the East China Normal University Animal Care Commission. All animals received humane care according to the criteria outlined in the Guide for the Care and Use of Laboratory Animals prepared by the National Academy of Sciences and published by the National Institutes of Health.

### Statistical analysis

All statistical analyses were done using SPSS 19.0 for windows (IBM Corporation) and GraphPad Prism 7 software (San Diego, CA). The correlation of LOXL4 expression with clinicopathologic parameters in patients with HCC was evaluated by the Chi-square test and Fisher’s exact probability method. Survival curves were calculated via the Kaplan-Meier method and analyzed by the log-rank test. Univariate and multivariate analyses were performed to identify the factors that had a significant influence on survival based on the Cox proportional hazards regression model. Other statistical data were represented as mean ± standard deviation (SD). Chi-square tests or Student’s t-tests were used for comparisons between groups. *P* < 0.05 was considered statistically significant.

## Results

### LOXL4 expression is significantly upregulated in HCC and is closely associated with clinicopathological features

**Fig. 1:**
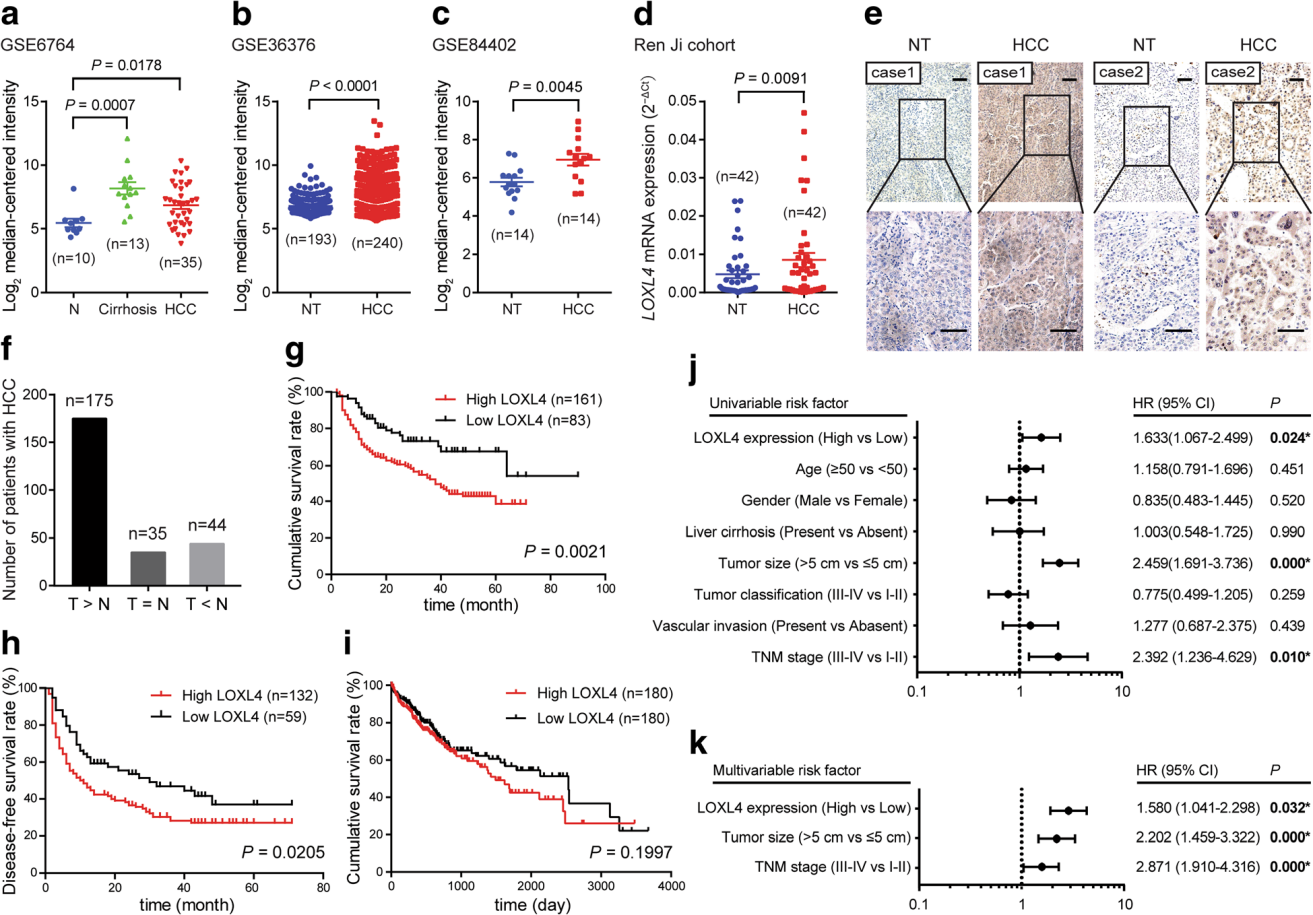
LOXL4 expression is upregulated in HCC tissues and predicts a poor clinical outcome. **a** The mRNA expression level of *LOXL4* in HCC tissues compared with the normal liver tissues (N) revealed using the GSE6764 dataset. **b-c** The mRNA expression level of *LOXL4* in the non-tumorous liver (NT) and HCC tissues revealed using the GSE36376 (**b**) and GSE84402 (**c**) datasets. **d** The mRNA expression level of *LOXL4* in 42 matched NT and HCC tissues derived from Ren Ji cohort detected by qRT-PCR. **e** IHC staining performed using an antibody against LOXL4 and representative photographs of the LOXL4 staining in NT and HCC tissues. (Scale bar: 100 μm). **f** LOXL4 expression was upregulated in 175 HCC tissues compared with NT tissues (T > N). **g** Comparison of overall survival of HCC patients with different LOXL4 protein expression. **h** Comparison of disease-free survival of HCC patients with different LOXL4 protein expression. **i** Overall survival analysis of HCC patients in TCGA cohort. **j-k** Forest plot showing the association between LOXL4 expression and HCC survival using univariate (**j**) and multivariate (**k**) analyses. (HR, hazard ratio; CI, confidence interval)

To illustrate the expression pattern of LOXL4 in HCC, we first evaluated the mRNA expression level of *LOXL4* by interrogating public gene expression databases. Data derived from GSE6764 set [26] showed that the expression of LOXL4 was significantly higher in HCC samples than that in normal liver samples (Fig. 1a). In another two independent datasets (GSE36376 [27] and GSE84402 [28]), we also observed a significantly higher expression of LOXL4 in HCC samples compared with their adjacent non-tumorous samples (Fig. 1b and c). In our cohort, similar result was also noticed in 42 paired HCC and non-tumorous tissues as demonstrated by qRT-PCR (Fig. 1d). Moreover, we performed IHC staining analysis in a tissue microarray containing 254 paired HCC and non-tumorous samples to detect LOXL4 protein expression. As a result, LOXL4 expression was upregulated in 175 out of 254 HCC cases (Fig. 1e and f).

To investigate the clinical significance of LOXL4 in HCC, we analyzed the association between LOXL4 expression and the clinicopathologic characteristics of HCC patients. As shown in Table 1, LOXL4 expression was significantly associated with differentiated grade, blood vessel invasion, and tumor-node-metastasis (TNM) stage, while there was no significant association between LOXL4 expression and age, gender, liver cirrhosis or tumor size.

**Table 1:** Correlations between LOXL4 expression and clinicopathologic parameters in patients with HCC

Clinicopathological parameter	N	Expression of LOXL4		*p* value^b^
		Low (*n* = 89, %)	High (*n* = 165, %)	
Age (years)				
< 50	120	45 (37.5)	75 (62.5)	0.259
≥ 50	134	44 (32.8)	90 (67.2)	
Gender				
Female	34	9 (26.5)	25 (73.5)	0.176
Male	220	80 (36.4)	140 (63.6)	
Liver cirrhosis				
Yes	201	68 (33.8)	133 (66.2)	0.264
No	53	21 (39.6)	32 (60.4)	
Tumor size				
≤ 5 cm	124	49 (39.5)	75 (60.5)	0.092
> 5 cm	130	40 (30.8)	90 (69.2)	
Tumor differentiation				
I	3	3 (100.0)	0 (0)	**0.003**
II	72	31 (43.1)	41 (56.9)	
III	125	31 (24.8)	94 (75.2)	
IV	1	0 (0)	1 (100.0)	
Vascular invasion				
Yes	58	12 (20.7)	46 (79.3)	**0.006**
No	196	77 (39.3)	119 (60.7)	
TNM stage				
I	125	49 (39.2)	76 (60.8)	**0.046**
II	56	24 (42.9)	32 (57.1)	
III	64	15 (23.4)	49 (76.6)	
IV	8	1 (12.5)	7 (87.5)	

^a^The bold number represents the *p*-values with significant differences

^b^*P* value was calculated by χ^2^ test or Fisher’s exact test

In addition, the correlation between LOXL4 expression and corresponding clinical follow-up information was analyzed by Kaplan-Meier curves and the log-rank test. High LOXL4 expression was found to be remarkably associated with decreased overall survival (Fig. 1g) and disease-free survival (Fig. 1h). The survival analysis of The Cancer Genome Atlas (TCGA) cohort showed the same trend, even if there was no statistical significance (Fig. 1i). Univariate and multivariate Cox regression analyses further confirmed LOXL4 expression as an independent predictor of the survival of patients with HCC in addition to tumor size and TNM stage (Fig. 1j and k). Taken together, these data indicate that upregulated LOXL4 has a significant correlation with poor prognosis of HCC as an independent prognostic factor and may contribute to HCC progression.

### LOXL4 expression is induced by TGF-β in HCC cells

**Fig. 2:**
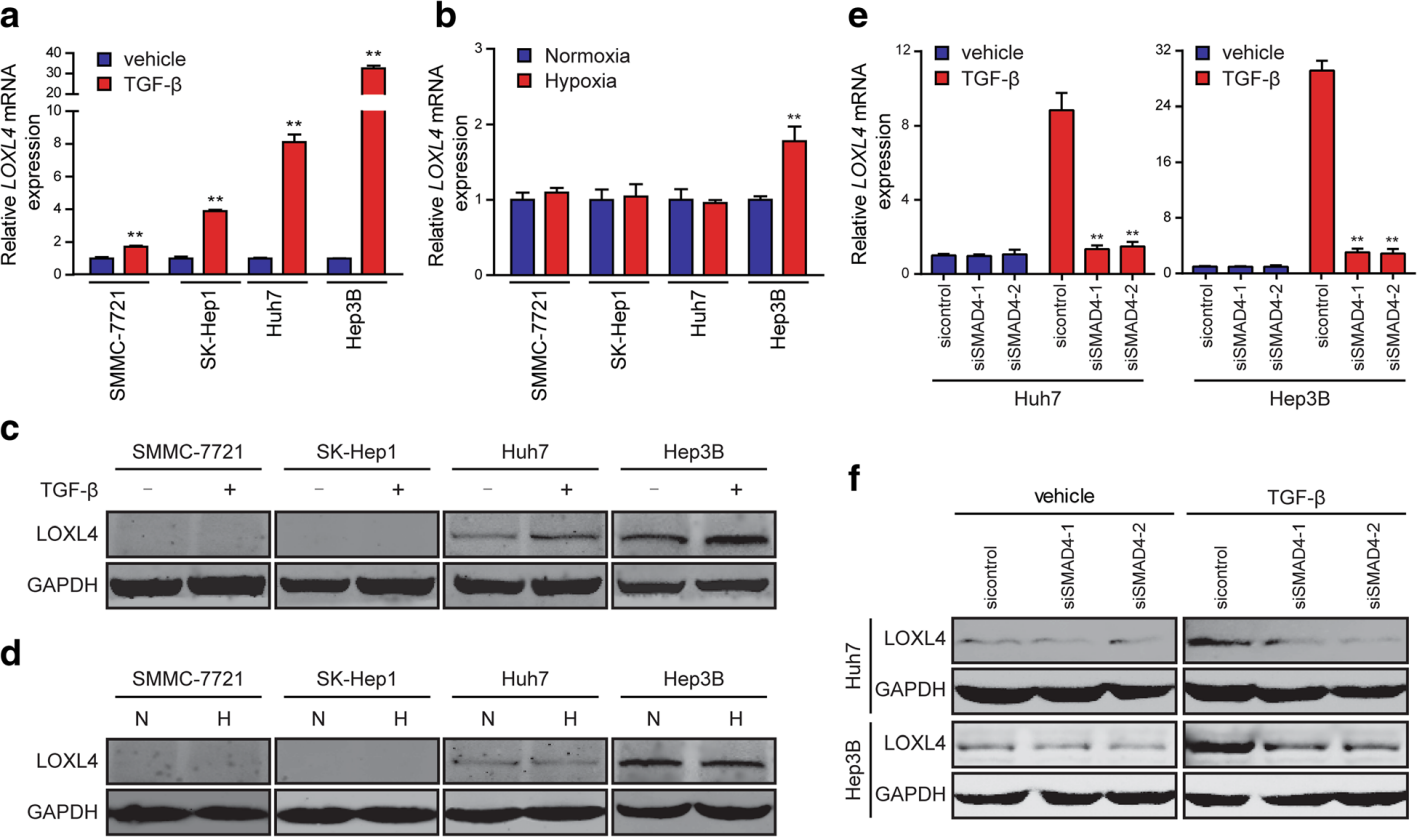
TGF-β induces LOXL4 expression in HCC cells. **a** *LOXL4* mRNA expression in HCC cells exposed to mock vehicle or TGF-β (5 ng/ml) for 12 h. **b** *LOXL4* mRNA expression in HCC cells exposed to 21% O_2_ (Normoxia) or 1% O_2_ (Hypoxia) for 16 h. **c** LOXL4 protein expression in HCC cells exposed to mock vehicle or TGF-β (5 ng/ml) for 12 h. **d** LOXL4 protein expression in HCC cells exposed to normoxia (N) or hypoxia (H) for 16 h. **e** *LOXL4* mRNA expression in Huh7 and Hep3B cells transfected with siRNAs targeting SMAD4 exposed to mock vehicle or TGF-β (5 ng/ml) for 12 h. **f** LOXL4 protein expression in Huh7 and Hep3B cells transfected with siRNAs targeting SMAD4 exposed to mock vehicle or TGF-β (5 ng/ml) for 12 h. (** *P* < 0.01)

Several studies have shown that LOXL4 expression is induced by TGF-β and hypoxia [29–34]. So, we then examined whether LOXL4 expression was induced by these two important parameters in the tumor microenvironment of HCC. We found that *LOXL4* expression was profoundly induced at mRNA level in multiple HCC cell lines (SMMC-7721, SK-Hep1, Huh7, and Hep3B) upon TGF-β treatment (Fig. 2a), but was profoundly induced at protein level only in Huh7 and Hep3B cells, rather than in SMMC-7721and SK-Hep1 cells, compared to vehicle treatment (Fig. 2c). However, hypoxia failed to induce LOXL4 expression compared to normoxia at both mRNA and protein levels (Fig. 2b and d).

To confirm that TGF-β induced LOXL4 expression through SMAD complex, SMAD4 siRNAs were transfected into Huh7 and Hep3B cells (Additional file 2: Figure S1). As shown in Fig. 2e and f, transfection with SMAD4 siRNAs abolished TGF-β-induced LOXL4 expression at both mRNA and protein levels in these two cell lines. These data indicate that TGF-β may be a critical regulator of LOXL4 expression in HCC.

### LOXL4 promotes cell migration and invasion in vitro and tumor metastasis in vivo

**Fig. 3:**
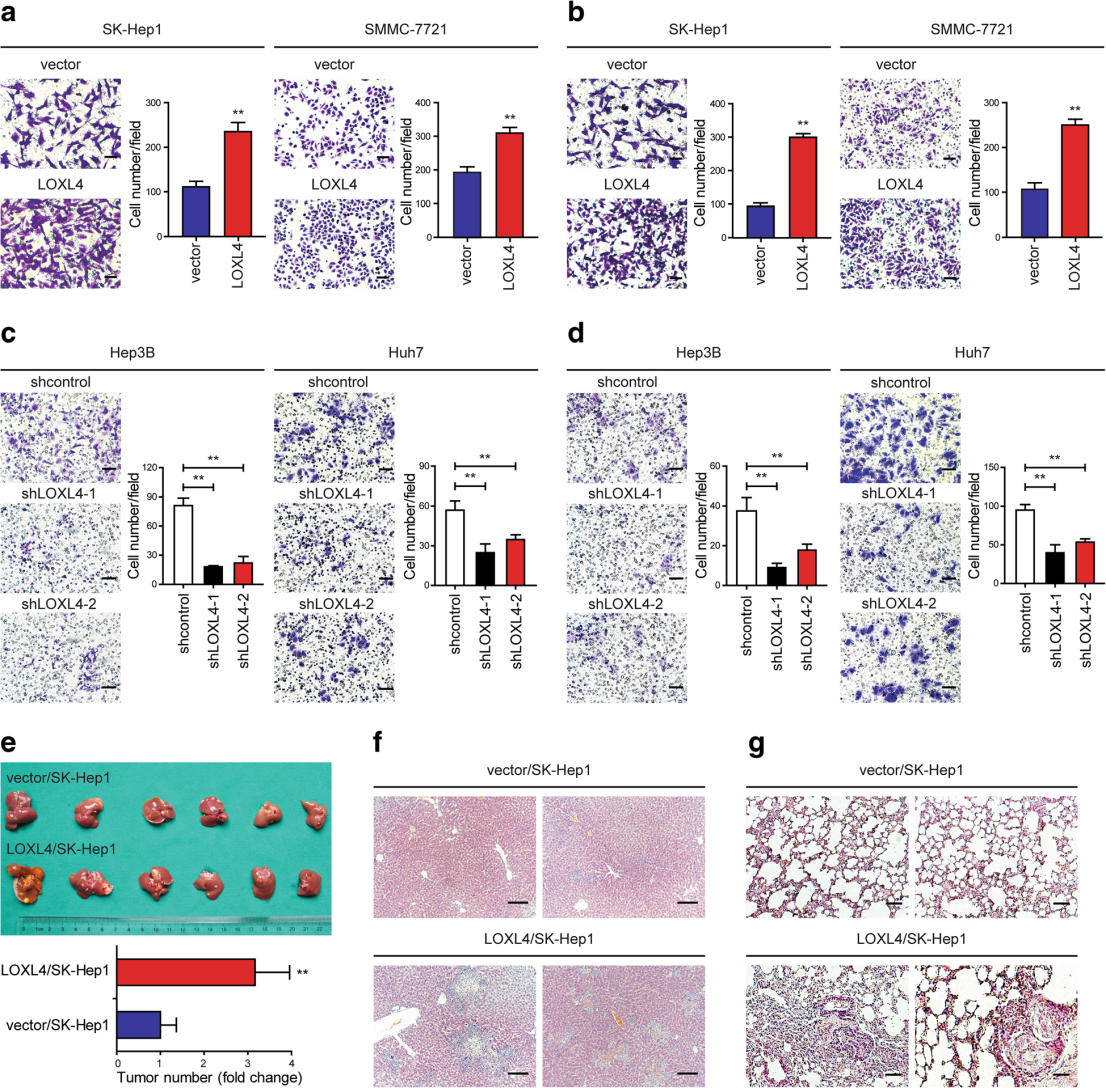
LOXL4 promotes HCC migration and invasion in vitro and metastasis in vivo. **a-b**. Cell migration (**a**) and invasion (**b**) potential was determined in SK-Hep1 and SMMC-7721 cells transfected with LOXL4 or control vector according to Transwell assays. **c-d**. Cell migration (**c**) and invasion (**d**) potential was determined in Hep3B and Huh7 cells transfected with shLOXL4 or shcontrol according to Transwell assays. **e** Tumor number on the liver surface 6 weeks after LOXL4-overexpressing SK-Hep1 cells (LOXL4/SK-Hep1) and control cells (vector/SK-Hep1) were orthotopically inoculated into the left hepatic lobe of nude mice. **f** Representative photographs of hematoxylin and eosin (H&E) staining in liver tissues from mice orthotopically inoculated with LOXL4/SK-Hep1 and control cells. **g** Lung metastases 6 weeks after LOXL4/SK-Hep1 and control cells were intravenously injected into nude mice detected by H&E staining. (** *P* < 0.01) (Scale bar: **a-d** 100 μm, **f-g** 200 μm)

The above observations promoted us to explore the functional role of LOXL4 in human HCC tumorigenesis and/or progression. Two cell lines with low LOXL4 expression, SK-Hep1 and SMMC-7721, were used for gain-of-function study (Additional file 2: Figure S2a and b). Stable transduction with lentivivus expressing LOXL4 resulted in drastic increases in LOXL4 expression at both mRNA and protein levels confirmed by qRT-PCR and western blotting, respectively (Additional file 2: Figure S3a and b). Among a series of cellular analyses, we found that the migratory and invasive capacity of SK-Hep1 and SMMC-7721 cells was significantly stimulated by LOXL4 overexpression, as revealed by Transwell assays (Fig. 3a and b). Anoikis is a special apoptotic process due to the loss of or inappropriate cell adhesion, leading to tumor recurrence and metastasis. We showed that LOXL4 overexpression significantly lowered the anoikis rate (Additional file 2: Figure S3c). However, LOXL4 overexpression had no significant implications on HCC cell proliferation (Additional file 2: Figure S3d).

To access the function of endogenous LOXL4 in HCC, we knocked down expression of LOXL4 using two LOXL4-specific shRNA lentiviruses (shLOXL4–1 or shLOXL4–2) in Hep3B and Huh7 cells, which expressed relatively higher LOXL4 (Additional file 2: Figure S2a and b). As controls, we used lentiviruses expressing nonspecific shRNA (shcontrol). Efficient LOXL4 knockdown was confirmed by qRT-PCR and western blotting, respectively (Additional file 2: Figure S4a and b). We found that cell migration and invasion were significantly inhibited in LOXL4 knockdown cells compared with control cells (Fig. 3c and d). Consistent with the observation of LOXL4 overexpression, cell proliferation was not affected by LOXL4 knockdown (Additional file 2: Figure S4c).

Next, to further explore the effect of LOXL4 on tumor metastasis in vivo, SK-Hep1 overexpressing LOXL4 (LOXL4/SK-Hep1) and control cells (vector/SK-Hep1) were orthotopically inoculated into the left hepatic lobe of mice or injected intravenously into nude mice. As expected, intrahepatic and lung metastases were frequently detected in the LOXL4/SK-Hep1 group but rarely in the vector/SK-Hep1 group (Fig. 3e-g), indicating that LOXL4 overexpression promoted intrahepatic and lung metastases of HCC in vivo.

### LOXL4 regulates cell-matrix adhesion via activating the FAK/Src signaling pathway in HCC cells

**Fig. 4:**
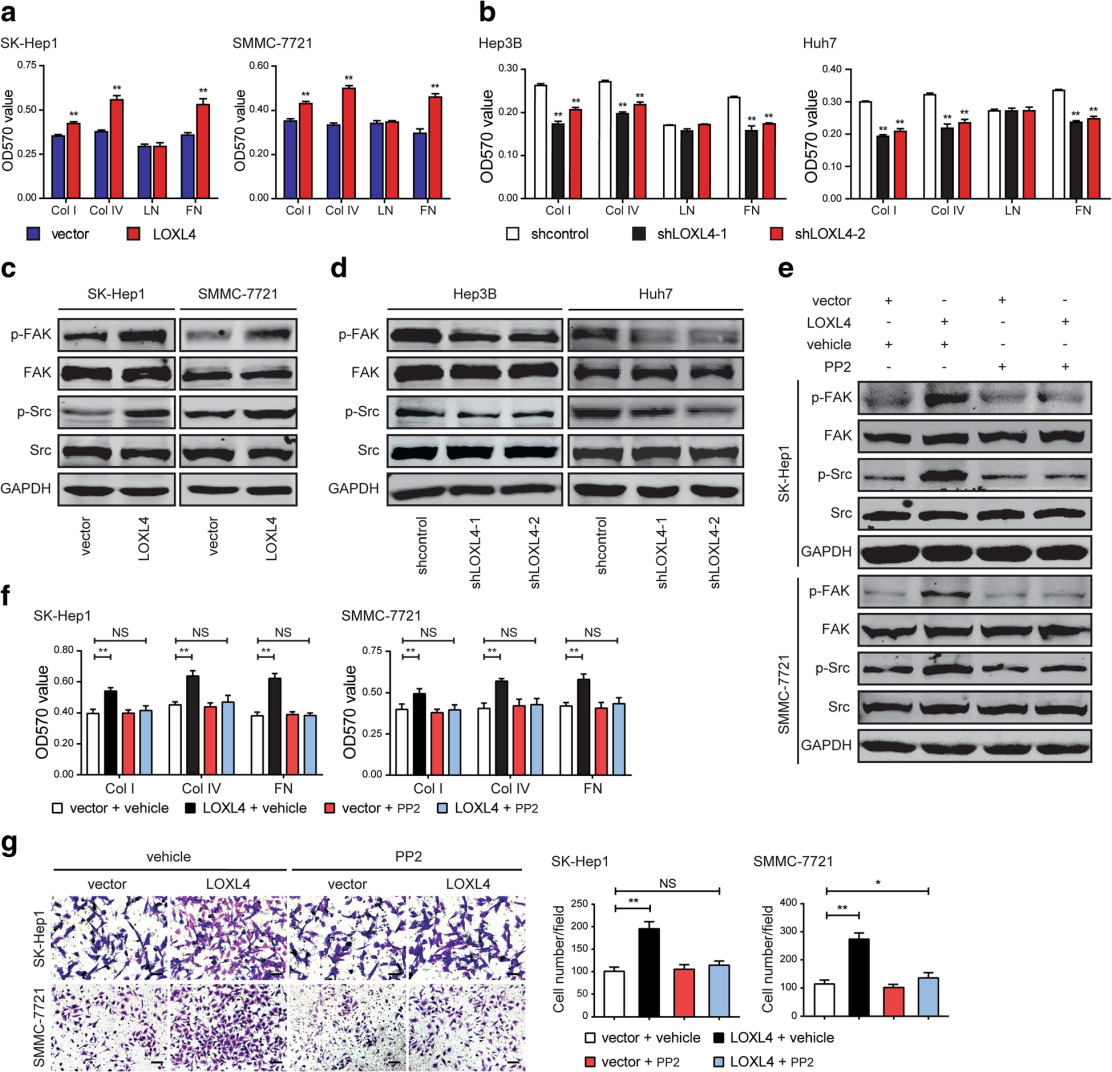
LOXL4 enhances cell-matrix adhesion and activates the FAK/Src pathway in HCC cells. **a** LOXL4-overexpressing and control cells were subjected to cell-matrix adhesion assay to collagen I (Col I), collagen IV (Col IV), laminin (LN), and fibronectin (FN). **b** LOXL4 knockdown and control cells were subjected to cell-matrix adhesion assay to Col I, Col IV, LN, and FN. **c** Western blotting analysis of phosphorylation of FAK and Src and total FAK and Src in LOXL4-overexpressing and control cells. **d** Western blotting analysis of phosphorylation of FAK and Src and total FAK and Src in LOXL4 knockdown and control cells. **e** Western blotting analysis of phosphorylation of FAK and Src and total FAK and Src in LOXL4-overexpressing and control cells upon treatment with vehicle or PP2 (10 μM) for 24 h. **f** LOXL4-overexpressing and control cells were subjected to cell-matrix adhesion assay to Col I, Col IV, and FN upon treatment with vehicle or PP2. **g** Cell migration potential was determined in LOXL4-overexpressing and control cells upon treatment with vehicle or PP2 according to Transwell assays (Scale bar: 100 μm). (* *P* < 0.05, ** *P* < 0.01)

To clearly uncover the reason for LOXL4-induced cell mobility, we measured cell-matrix adhesion in HCC cells. The results showed that LOXL4 overexpression led to a significant increase in cell adhesion to collagen I, collagen IV, and fibronectin (Fig. 4a). Consistently, LOXL4 knockdown had the opposite effects (Fig. 4b). However, neither overexpression nor knockdown of LOXL4 affected cell adhesion to laminin. Collectively, LOXL4 facilitates cell-matrix adhesion in HCC cells.

Our previous study has showed that LOXL4 promotes proliferation and metastasis of gastric cancer via activating the FAK/Src pathway [11]. Cell-matrix adhesion was altered by LOXL4 in HCC cells, and this process is regulated by two key proteins: FAK and Src [35, 36], which are activated through a series of phosphorylation events. Therefore, in this study, we examined the FAK/Src pathway as a candidate regulated by LOXL4 in HCC cells. We observed that overexpression of LOXL4 markedly increased the phosphorylation levels of FAK and Src (Fig. 4c), while shRNA-mediated knockdown of LOXL4 gave rise to a significant decrease in the phosphorylation of FAK and Src (Fig. 4d).

To further illuminate that LOXL4 regulated cell-matrix adhesion and cell migration via the FAK/Src pathway, we treated HCC cells with the Src inhibitor PP2. As a result, PP2 significantly suppressed Src and FAK activation (Fig. 4e), cell-matrix adhesion (Fig. 4f), and cell migration (Fig. 4g) induced by LOXL4 overexpression. These data indicate that LOXL4-mediated cell-matrix adhesion and cell migration in HCC is via upregulation of Src and FAK phosphorylation.

### LOX catalytic activity is indispensable for LOXL4 to promote cell migration and activate the FAK/Src pathway through a hydrogen peroxide-mediated mechanism

Lysyl oxidases function as inducers of tumor cell invasiveness, which are found to be dependent on their catalytic activity [36–42]. LOX activity has been reported to regulate tumor cell behaviors by oxidizing lysine residues of their substrates to produce hydrogen peroxide, and it has recently been shown that the presence of hydrogen peroxide can facilitate Src activation [30, 33, 36]. Therefore, we hypothesized that LOXL4 promoted cell-matrix adhesion dependent on its catalytic activity by activating the FAK/Src pathway through a hydrogen peroxide-mediated mechanism.

**Fig. 5:**
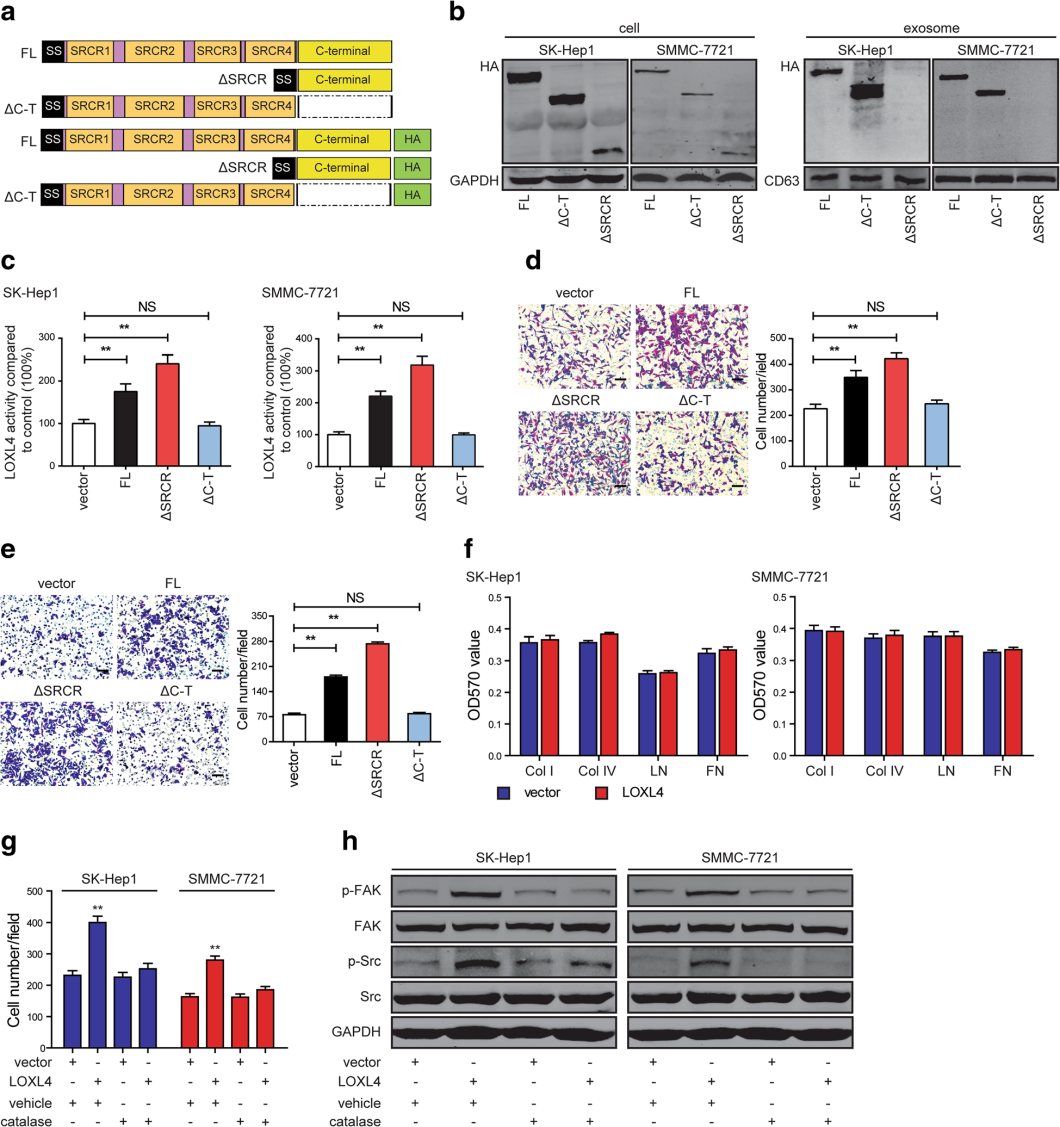
LOX catalytic activity is responsible for LOXL4 to promote cell-matrix adhesion and cell migration. **a** Domain structure of wild-type LOXL4 protein (full-length, FL) and deletion mutants thereof lacking the SRCR domains (ΔSRCR) and the C-terminal catalytic region (ΔC-T) with or without the HA-tag. **b** Analysis of the LOXL4-HA chimeras stably expressed in HCC cells and their corresponding exosomes by western blotting using an anti-HA antibody. **c** Intracellular LOXL4 catalytic activity measured in whole-cell lysates of control, FL, and deletion mutant cells. **d-e** Cell migration potential was determined in SK-Hep1 (**d**) and SMMC-7721 (**e**) cells transfected with FL, deletion mutants or control vector according to Transwell assays (Scale bar: 100 μm). **f** LOXL4-overexpressing and control cells were subjected to cell-matrix adhesion assay to Col I, Col IV, LN, and FN with catalase treatment (500 U/ml) for 12 h. **g** Cell migration potential was determined in LOXL4-overexpressing and control cells upon treatment with vehicle or catalase according to Transwell assays. **h** Western blotting analysis of phosphorylation of FAK and Src and total FAK and Src in LOXL4-overexpressing and control cells with vehicle or catalase treatment. (** *P* < 0.01)

To confirm the hypothesis, we first examined whether the catalytic activity is required for LOXL4 to promote cell-matrix adhesion and cell migration. We constructed the LOXL4 wild-type and deletion mutants with or without the HA-tag simultaneously as depicted (Fig. 5a), which were applied to transfect SK-Hep1 and SMMC-7721 cells, and their expression levels were examined in HCC cells (Fig. 5b left panel). Subsequently, intracellular LOX catalytic activity was measured in these cells and showed that LOXL4 wild-type (full-length, FL) and LOXL4-lacking SRCR domains (ΔSRCR) cells had a 2- to 4-fold increase compared with control cells, while LOXL4-lacking the entire catalytic domain (ΔC-T) cells had a similar level to control cells (Fig. 5c). We found that LOXL4 deletion mutant ΔC-T had no effects on cell migration (Fig. 5d and e), indicating that the catalytic activity is necessary for LOXL4 to function. However, LOXL4 deletion mutant ΔSRCR had a higher catalytic activity and significantly promoted cell migration compared to LOXL4 wild-type (Fig. 5d and e).

Then catalase, an enzyme responsible for converting hydrogen peroxide to water and molecular oxygen, was used to examine the ability of hydrogen peroxide to regulate cell-matrix adhesion, cell migration, and Src activation. We found that catalase completely abolished LOXL4-induced increases in cell-matrix adhesion (Fig. 5f) and cell migration (Fig. 5g). Consistently, catalase completely abolished shLOXL4-induced decreases in cell-matrix adhesion and cell migration (Additional file 2: Figure S5a and b). Finally, catalase completely abolished the effects of LOXL4 on the FAK/Src pathway in HCC cells (Fig. 5h, Additional file 2: Figure S5c). Taken together, we show that LOXL4 promotes cell-matrix adhesion and cell migration dependent on its catalytic activity by activating the FAK/Src pathway through a hydrogen peroxide-mediated mechanism.

### Secreted LOXL4 but not rhLOXL4 promotes HCC migration

**Fig. 6:**
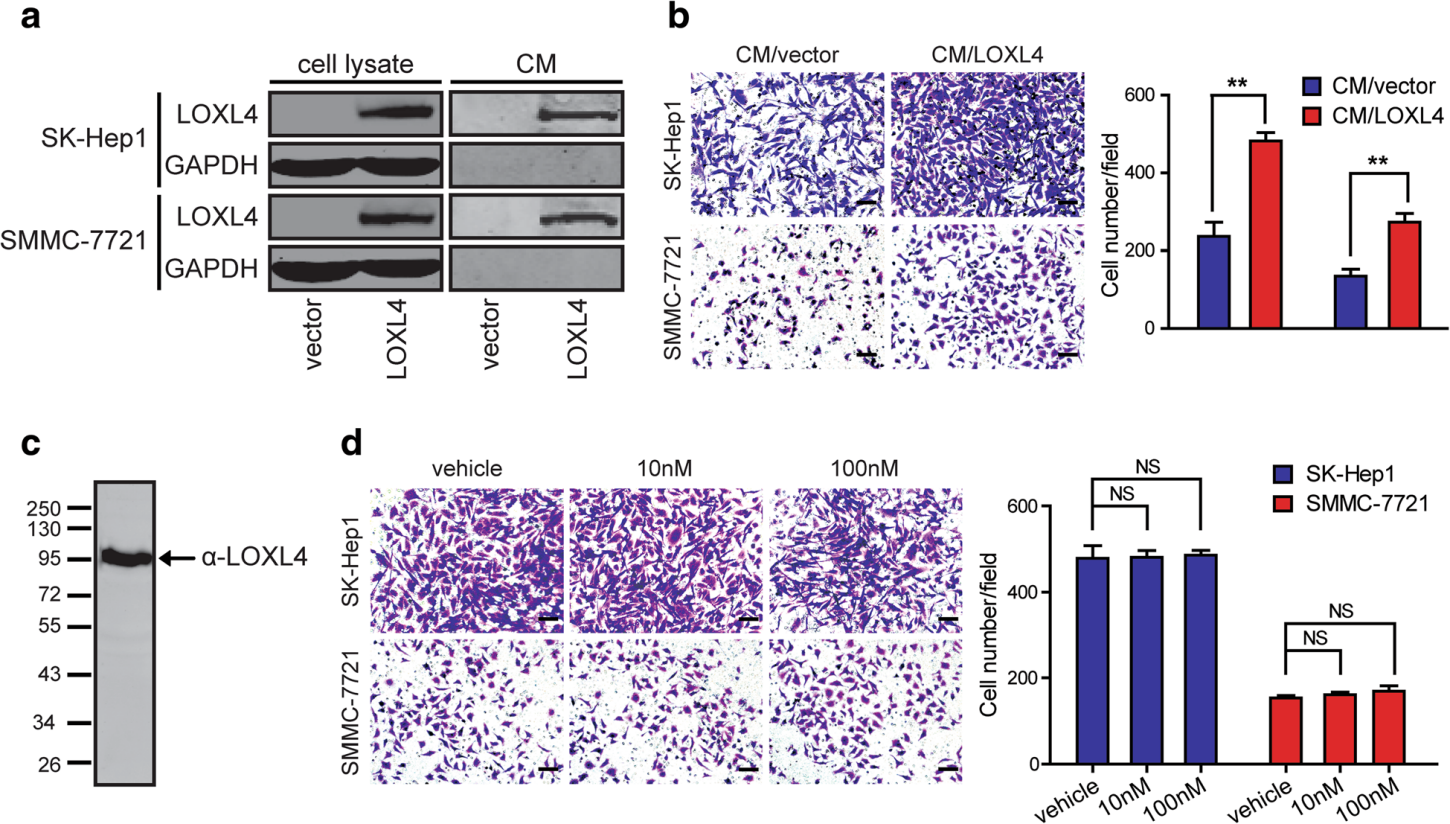
Secreted LOXL4 but not rhLOXL4 promotes HCC migration. **a** Examination of LOXL4 protein in the CM derived from LOXL4-overexpressing cells (CM/LOXL4) and control cells (CM/vector) by western blotting. **b** Cell migration potential was determined in parental SK-Hep1 and SMMC-7721 cells incubated with CM/LOXL4 and CM/vector according to Transwell assays. **c** Identification of rhLOXL4 secreted by human embryonic kidney 293/Epstein-Barr nuclear antigen cells by western blotting with an antibody against LOXL4. **d** Cell migration potential was determined in SK-Hep1 and SMMC-7721 cells upon treatment with vehicle or different concentrations of rhLOXL4 according to Transwell assays. (Scale bar: **b** and **d** 100 μm). (** *P* < 0.01)

LOXL4 is an important extracellular matrix protein, and therefore, we examined whether LOXL4 protein is secreted by HCC cells into the culture supernatant. All the cell lines used for LOXL4 overexpression and knockdown study were subjected to standardized conditions to collect CM, and the secreted LOXL4 proteins were detectable only in the CM from LOXL4-overexpressing cells (Fig. 6a) and shcontrol cells (Additional file 2: Figure S6) by western blotting, suggesting that both exogenous and endogenous LOXL4 can be secreted into CM. To evaluate the function of secreted LOXL4 in HCC cell migration, SK-Hep1 and SMMC-7721, two cell lines with low basal levels of LOXL4, were chosen to undergo Transwell migration assays in the presence of CM derived from LOXL4-overexpressing (CM/LOXL4) and control cells (CM/vector). The results showed that CM/LOXL4 significantly promoted the migration of SK-Hep1 and SMMC-7721, compared to CM/vector (Fig. 6b), suggesting the possible existence of a mechanism able to transduce LOXL4 signals from the extracellular space into the cells.

To investigate whether secreted LOXL4 directly promotes HCC migration, a eukaryotic expression system suitable for secreted protein synthesis was used to generate rhLOXL4 with high fidelity (Fig. 6c). Unexpectedly, the migration of SK-Hep1 and SMMC-7721 cells was not affected by rhLOXL4 (Fig. 6d). The results suggested that intracellular LOXL4 promoted HCC cell migration rather than extracellular LOXL4, and that secreted LOXL4 in CM may again enter HCC cells independently of a cell membrane-anchored LOXL4 receptor to exert its function. Thus, we speculated that secreted LOXL4 proteins were likely to be present in a particle-associated form and could be internalized by HCC cells to function intracellularly.

### Intercellular transfer of LOXL4 by exosomes between HCC cells to promote HCC migration

**Fig. 7:**
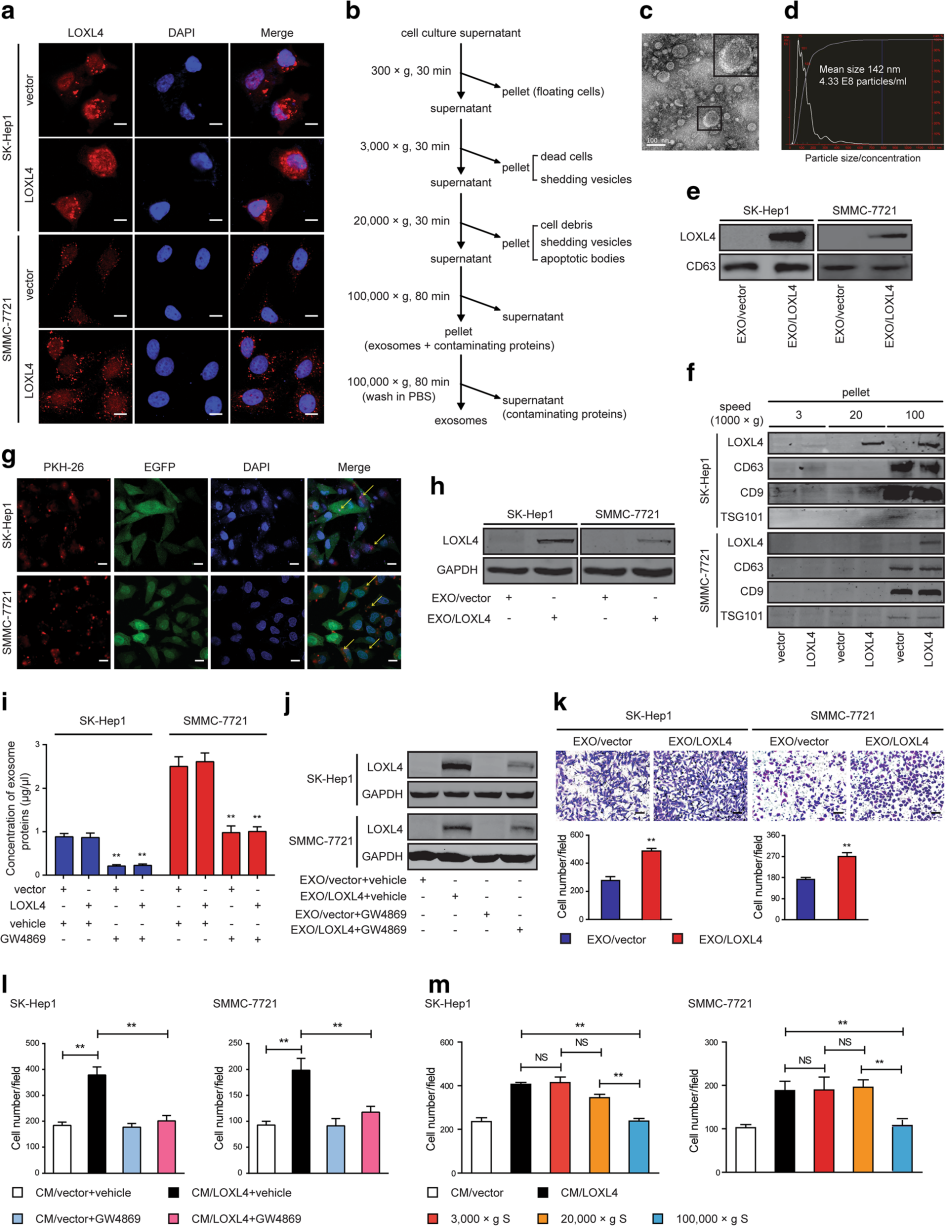
Intercellular transfer of LOXL4 by exosomes though autocrine/paracrine mechanisms to promote HCC migration. **a** IF images of LOXL4 in LOXL4-overexpressing and control cells. Red: LOXL4; Blue: DAPI (nuclei) (Scale bar: 100 μm). **b** Flow chart for the exosome purification procedure based on differential ultracentrifugation. **c**. electron microscopy of purified exosomes derived from SK-Hep1 cells overexpressing LOXL4 (Scale bar: 100 nm). **d** Characterization of purified exosomes derived from SK-Hep1 cells overexpressing LOXL4. Assessment of size, number, and distribution by NTA technology. **e** Western blotting analysis of exosomal marker CD63 and LOXL4 in exosomes purified from LOXL4-overexpressing cells (EXO/LOXL4) and control cells (EXO/vector). **f** Western blotting analysis of the corresponding pellets produced by differential ultracentrifugation for CD63, CD9, TSG101 (exosome marker proteins) and LOXL4. **g** Parental HCC cells expressing EGFP were incubated with PKH26-labelled EXO/LOXL4 for 2 h, and the arrows indicate exosomes incorporated into HCC cells. (Scale bar: 100 μm). **h** Detection of LOXL4 in parental HCC cells incubated with EXO/LOXL4 and EXO/vector by western blotting. **i** The effect of GW4869 (5 μM, 72 h) on the concentration of exosomes measured by BCA protein assay. **j** Detection of LOXL4 in parental HCC cells treated with exosomes derived from LOXL4-overexpressing and control cells incubated with vehicle or GW4869. **k** Cell migration potential was determined in SK-Hep1 and SMMC-7721 cells upon treatment with EXO/vector or EXO/LOXL4 (50 μg/ml) (Scale bar: 100 μm). **l** Cell migration potential was determined in SK-Hep1 and SMMC-7721 cells upon treatment with the CM from LOXL4-overexpressing and control cells incubated with vehicle or GW4869. **m** Cell migration potential was determined in SK-Hep1 and SMMC-7721 cells upon treatment with supernatant acquired by differential ultracentrifugation (supernatant, S). (** *P* < 0.01)

LOXL4 has both intracellular and secreted forms, which was further confirmed by the IF analysis (Fig. 7a) and western blotting (Fig. 6a). We observed scattered immunofluorescent particles in the cytoplasm of both LOXL4-overexpressing and control cells (Fig. 7a), suggesting that both endogenous and exogenous LOXL4 may exist in cystic organelles, such as exosomes. Analysis of ExoCarta (http://www.exocarta.org), an exosome database, revealed that LOXL4 resides in exosomes derived from different cell types, in addition to LOX, LOXL2, and LOXL3 (Additional file 2: Figure S7a). Exosomes purified from cell culture supernatant of HCC cells by differential centrifugation (Fig. 7b) exhibited a typical cup-shaped morphology and a size range of 30 to 150 nm according to electron microscopy and NTA (Fig. 7c and d). These vesicles were further confirmed by immunostaining for the well-defined exosome markers CD63, CD9, and TSG101 (Fig. 7e and f). The results also revealed that exosomal fractions from HCC cells contained LOXL4 that mirrored the intracellular levels of LOXL4. Interestingly, we found that 20,000×g pellets derived from SK-Hep1/LOXL4 cells also contained LOXL4 (Fig. 7f), suggesting that LOXL4 existed in both exosomes and larger-sized microvesicles.

We had showed the role of intracellular LOXL4 in HCC progression through the modulation of migration, invasion, and metastasis by gain- and loss-of-function studies (Fig. 3). Therefore, we sought to determine whether LOXL4-containing exosomes can be internalized by HCC cells to influence their migratory response. To this end, HCC cells expressing enhanced green fluorescent protein (EGFP) were incubated with exosomes labeled with PKH26, and the confocal microscopy results showed that HCC cells exhibited high uptake efficiency (Fig. 7g). Next, to confirm that HCC-secreted LOXL4 can be horizontally transferred between HCC cells via exosomes, we measured the LOXL4 levels in parental SK-Hep1 and SMMC-7721 cells treated with exosomes derived from LOXL4-overexpressing (EXO/LOXL4) and control cells (EXO/vector). An increase in LOXL4 was observed in recipient HCC cells following treatment with EXO/LOXL4 (Fig. 7h); however, there was no change in the mRNA levels of *LOXL4* in these cells (Additional file 2: Figure S7b). Furthermore, GW4869, an N-SMase inhibitor that pharmacologically blocks exosome generation, was utilized to block HCC exosome release and confirm exosome-mediated transfer of LOXL4 between HCC cells. Expectedly, we found that GW4869 successfully decreased exosome secretion in both LOXL4-overexpressing and control cells (Fig. 7i) and observed a marked decrease of LOXL4 in recipient HCC cells treated with exosomes derived from LOXL4-overexpressing cells incubated with GW4869 (EXO/LOXL4 + GW4869) (Fig. 7j), further indicating that LOXL4 was horizontally transferred between HCC cells via exosomes.

Next, Transwell migration assays were performed to determine whether LOXL4-containing exosomes internalized by HCC cells are sufficient to induce cell migration. We found that HCC cell migration was significantly stimulated by EXO/LOXL4 compared with EXO/vector (Fig. 7k). In contrast, depletion of exosomes, using GW4869 treatment or centrifugation at 100,000×g, dramatically attenuated the stimulatory activity in the medium from LOXL4-overexpressing cells (Fig. 7l and m), revealing that LOXL4-containing exosomes possessed CM stimulatory activity. Taken together, these data show that the unique LOXL4 content of HCC-derived exosomes functions as a migratory regulator in HCC cells.

### Intercellular transfer of LOXL4 by exosomes to HUVECs to promote angiogenesis

**Fig. 8:**
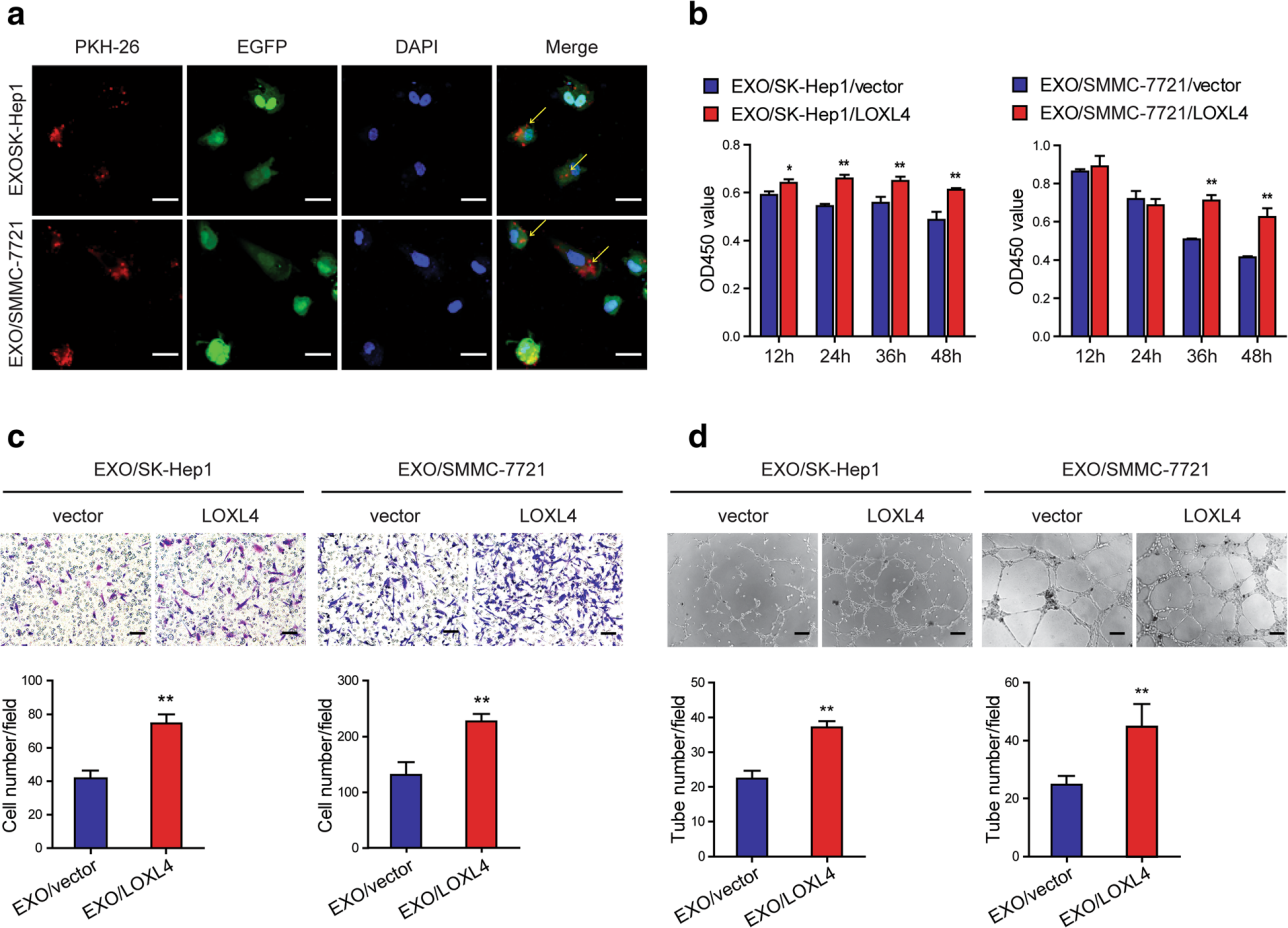
Intercellular transfer of LOXL4 by exosomes to HUVECs to promote angiogenesis. **a** HUVECs expressing EGFP incorporated PKH26-labelled EXO/LOXL4, and the arrows indicate exosomes incorporated into HUVECs (Scale bar: 20 μm). **b** Cell viability was measured by CCK-8 assay in HUVECs treated with EXO/vector and EXO/LOXL4 (50 μg/ml). **c** Cell migration potential was determined in HUVECs treated with EXO/vector and EXO/LOXL4 (Scale bar: 100 μm). **d** HUVEC tuber formation in the present of EXO/vector or EXO/LOXL4 (Scale bar: 100 μm). (* *P* < 0.05, ** *P* < 0.01)

Tumor-derived exosomes can influence cancer progression and metastasis by the transfer of bioactive molecules between different cell types in the tumor microenvironment [43]. Several studies have shown that LOX and LOXL2 promote angiogenesis to drive tumor metastasis [44–47]. To confirm that HCC-derived LOXL4 can be transferred to endothelial cells via exosomes to promote angiogenesis, PKH26 labeled exosomes were incubated with HUVECs expressing EGFP. We observed the incorporation of exosomes into HUVECs by confocal microscopy (Fig. 8a), and an increase of LOXL4 in recipient HUVECs following the treatment with EXO/LOXL4 (Additional file 2: Figure S8a). Similarly, a decrease of LOXL4 was also observed in recipient HUVECs treated with EXO/LOXL4 + GW4869 (Additional file 2: Figure S8b). We concluded that this increase of LOXL4 reflects the exosome-mediated protein transfer but not an induction of endogenous expression in the recipient cells, as the *LOXL4* mRNA level in exosome-treated cells was not significantly changed (Additional file 2: Figure S8c). To investigate whether LOXL4 delivered by HCC-derived exosomes can affect the behaviors of HUVECs, a series of cellular analyses were performed. The results showed that EXO/LOXL4 enhanced the HUVEC survival (Fig. 8b), migration (Fig. 8c), and tube formation (Fig. 8d). The results demonstrate that HCC-derived exosomes transfer LOXL4 proteins to HUVECs though a paracrine mechanism to promote angiogenesis.

## Discussion

The development of HCC is a multistep process that advances from chronic HBV/HCV infection, fibrosis, cirrhosis, dysplasia/dysplastic nodules, and early HCC to metastatic HCC [48]. In the case of HCC, chronic HBV/HCV infection causes liver damage and inflammation, resulting in an increase in TGF-β, which was shown to induce expression of LOXL4 in HCC cells in our study. Our data are supported by several studies that TGF-β can induce the expression of LOXL4 in various cell types, such as human trabecular meshwork cells [29], myeloid cells [30, 31] and aortic endothelial cells [32]. A great number of studies have confirmed that LOX family play important roles in liver fibrosis and cirrhosis, and online datamining revealed that LOXL4 expression was significantly increased in cirrhosis tissues, even than that in HCC tissues (Fig. 1a), suggesting that TGF-β-induced LOXL4 plays important roles in multisteps of HCC development, especially in the process of liver fibrosis and cirrhosis.

Other researchers and we have demonstrated that the expression of LOXL4 is upregulated in several human cancers, including head and neck squamous cell carcinoma (HNSCC) [47], laryngeal squamous cell carcinoma [49], and gastric cancer [11]. Others have shown that LOXL2 is frequently overexpressed in human HCC [50, 51], and we showed that LOXL4 expression was also increased at both mRNA and protein levels in HCC. Furthermore, we found that LOXL4 upregulation was significantly correlated with tumor differentiation, vascular invasion, and TNM stage and predicted a poor prognosis, in contrast to a previous study demonstrating that LOXL4 is downregulated in HCC tissues, and the downregulation is correlated with aggressive tumors and a worse clinical outcome [12].

The mode of catalytic activity is believed to be consistent in all LOX and LOXL proteins [52], and accumulating studies have shown that LOX activity plays important roles in tumor invasion and metastasis. Consistent with previous studies, we found that LOXL4 significantly promoted cell adhesion, migration, and invasion in vitro and tumor metastasis in vivo via gain- and loss-of-function studies and provide strong evidence that LOXL4 is a key determinant of HCC metastasis. In addition, LOX catalytic activity was also shown to be indispensable for LOXL4 to promote HCC cell migration via construction of deletion mutants and depletion of hydrogen peroxide via catalase treatment. Relevant to our study, LOX activity has been reported to promote tumor migration, invasion, and metastasis via induction of the epithelial-mesenchymal transition (EMT) [38, 53–56] or activation of Src and FAK to regulate tumor behaviors through hydrogen peroxide, a byproduct produced in the process of LOX oxidizing the lysine residues of their substrates [57–60]. In our study, we did not observe the EMT phenomenon in LOXL4-overexpressing cells (data not shown), suggesting that LOXL4 does not promote tumor metastasis via triggering the EMT process; however, we found that LOXL4 contributed to hydrogen peroxide-mediated activation of the FAK/Src pathway and thereby promoted HCC cell adhesion to extracellular matrix (ECM) components collagen I, collagen IV, and fibronectin.

Tumor cell to form a stable adhesion to ECM is a key process of tumor cell invasion, including attachment to ECM, degradation of ECM, detachment from ECM, and finally active migration from the primary tumor [61]. Cell adhesion is regulated by two key proteins FAK and Src within cells, which have been demonstrated to be activated by LOXL4 in our study. In addition, the FAK/Src pathway is a well-documented pro-survival pathway in anoikis resistance [62]. Acquisition of anoikis resistance is a prerequisite for intra-hepatic spreading and extra-hepatic metastasis of HCC. We found that LOXL4 overexpression enhanced anoikis resistance. These data collectively demonstrate that LOXL4 plays important roles in HCC cell invasion process, not only in cell attachment to ECM but also in pro-survival after cell detachment from ECM.

LOXL4 is an important extracellular protein that can be secreted into the ECM. A recent study demonstrates that the LOXL4-mAb has potent antitumor activity for therapeutic targeting of HNSCC cells and xenografts [13]. However, in our study, rhLOXL4 had no effect on cell migration, suggesting that intracellular LOXL4 exerts its function in promoting HCC cell migration, consistent with the observation that positive immunostaining for LOXL4 is predominantly observed in the cytoplasm of tumor cells (Fig. 1e). However, the CM secreted by LOXL4-overexpressing cells could promote cell migration. We speculated that LOXL4 may be internalized though some carriers to function intracellularly, or tumor metastasis-related factors induced by LOXL4 secreted into the CM may promote cell migration. Intriguingly, it has been reported that LOXL4 can be detected in exosomes derived from human parotid glands [63], bovine milk [64] and rat adipocytes [65], reminding us that LOXL4 may be secreted into CM in an exosome-dependent manner. Our study, for the first time, provides a proof of principle that LOXL4 resides in exosomes derived from HCC cells based on IF analysis and western blotting. Simultaneously, we found that SRCR domain deletion mutant was not detectable in exosomes (Fig. 6b right panel), suggesting that the SRCR domains may play an important role in the process of LOXL4 packaging into exosomes at least in the context of HCC. However, how LOXL4 is packaged into exosomes warrants further investigation.

The exchange of cellular materials between cells through various autocrine, paracrine, and endocrine mechanisms is important for intercellular communication and can be mediated by exosomes. Intensive research has described the role of tumor-derived exosomes in tumor progression and aggressiveness [66]. Our data indicated that HCC-derived exosomes can transfer LOXL4 to parental HCC cells and endothelial cells, thereby enhancing the invasive potential of HCC cells, promoting angiogenesis, and ultimately facilitating HCC metastasis. These results suggest that the secreted LOXL4-containing exosomes in the extracellular environment may largely serve as autocrine/paracrine stimuli to promote HCC metastasis in a heterogeneous tumor cell population or via tumor-stromal cell interactions. It was not evaluated in this study how HCC-derived exosomes are internalized by HCC and endothelial cells. Recently, it has been reported that endothelial cell-derived exosomes mediate ECM crosslinking by upregulation of LOXL2 under hypoxic conditions [67], implying that it would be of interest to determine the noncancer source of LOXL4 carried by exosomes in tumor microenvironment and its role in regulating tumor metastasis through the herein demonstrated mechanisms in HCC.

## Conclusions

**Fig. 9:**
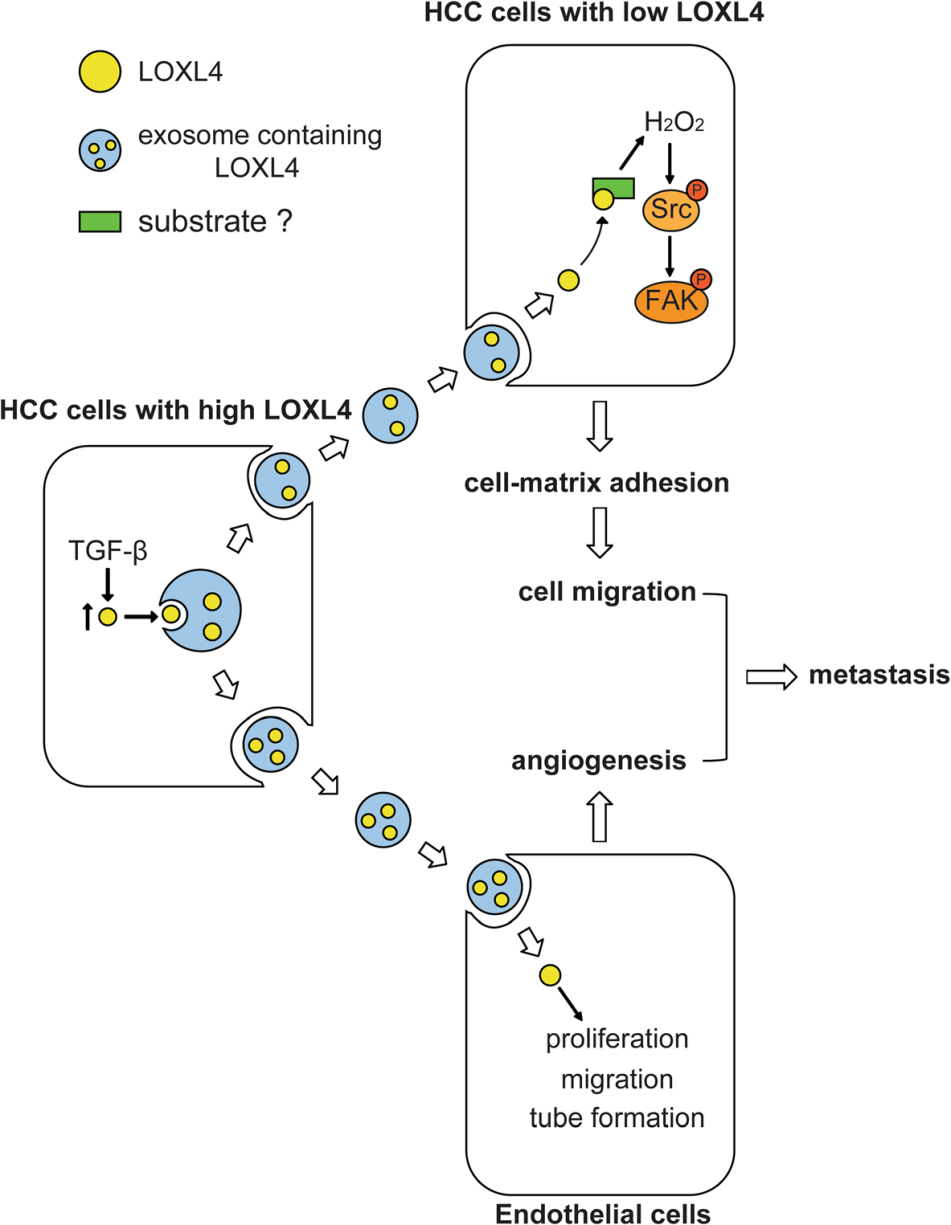
Mechanisms by which exosome-mediated secretion of LOXL4 promotes hepatocellular carcinoma cell invasion and metastasis. (H_2_O_2_, hydrogen peroxide)

In conclusion, to our knowledge, we showed for the first time that LOXL4 can be transferred between HCC cells with different expression levels of LOXL4 via exosomes and that intracellular LOXL4 promotes HCC motility and metastasis by activating the FAK/Src pathway dependent on its amine oxidase activity through a hydrogen peroxide-mediated mechanism; in addition, exosomes derived from HCC cells can transfer LOXL4 to HUVECs to promote angiogenesis via maintaining cell survival, promoting cell migration, and inducing tube formation (Fig. 9). These data provide substantial new evidence that LOXL4 is involved in cancer metastasis and indicate that LOXL4 may serve as a novel prognostic marker and therapeutic target for HCC.

## Additional files

Additional file 1: **Table S1.** Primers for generation of LOXL4 deletion mutants. (DOCX 15 kb)

Additional file 2: **Figure S1.** The silencing efficacy of SMAD4 siRNAs in Huh7 and Hep3B cells was examined by western blotting. **Figure S2.** LOXL4 expression in HCC cell lines. a. *LOXL4* expression at mRNA level in HCC cell lines examined by qRT-PCR. 18sRNA was detected as an internal control. b. LOXL4 expression at protein level in HCC cell lines examined by western blotting. **Figure S3.** Validation of LOXL4 overexpression and the effects of LOXL4 overexpression on cell proliferation and anoikis. a. QRT-PCR analysis of *LOXL4* expression in LOXL4-overexpressing and control cells. b. Western blotting analysis of LOXL4 expression in LOXL4-overexpressing and control cells. c. Annexin V/PI stain by FACS was performed to measure anoikis rate after overexpression of LOXL4. d. Cell viability was measured by CCK-8 assay after overexpression of LOXL4. (** *P* < 0.01). **Figure S4.** Validation of LOXL4 knockdown and the effects of LOXL4 knockdown on cell proliferation. a. QRT-PCR analysis of *LOXL4* expression in LOXL4 knockdown and control cells. b. Western blotting analysis of LOXL4 expression in LOXL4 knockdown and control cells. c. Cell viability was measured by CCK-8 assay after knockdown of LOXL4. **Figure S5.** The effects of LOXL4 knockdown on cell-matrix adhesion and the FAK/Src pathway are completely abolished by catalase. a. LOXL4 knockdown and control cells were subjected to cell-matrix adhesion assay to Col I, Col IV, LN, and FN with catalase treatment (500 U/ml) for 12 h. b. Cell migration potential was determined in LOXL4 knockdown and control cells upon treatment with vehicle or catalase according to Transwell assays. c. Western blotting analysis of phosphorylation of FAK and Src and total FAK and Src in LOXL4 knockdown and control cells with catalase treatment. (** *P* < 0.01). **Figure S6.** Examination of LOXL4 protein in the CM derived from LOXL4 knockdown cells and control cells by western blotting. **Figure S7.** a. Query results for gene symbol LOX in ExoCarta. b. The mRNA level of *LOXL4* detected by qRT-PCR in parental SK-Hep1 and SMMC-7721 cells treated with EXO/vector and EXO/LOXL4. **Figure S8.** Examination of LOXL4 in HUVECs treated with exosomes derived from HCC cells. a. LOXL4 protein expression was detected by western blotting in HUVECs treated with exosomes derived from HCC cells. b. LOXL4 protein expression was detected by western blotting in HUVECs treated with exosomes derived from HCC cells incubated with vehicle or GW4869. c. *LOXL4* mRNA expression was detected by qRT-PCR in HUVECs treated with exosomes derived from HCC cells. (ZIP 7026 kb)

## Abbreviations

CM: Conditioned media
ECM: Extracellular matrix
HCC: Hepatocellular carcinoma
HNSCC: Head and neck squamous cell carcinoma
HUVECs: Human umbilical vein endothelial cells
IF: Immunofluorescence
IHC: Immunohistochemical
LOX: Lysyl oxidase
LOXL4: Lysyl oxidase-like 4
MVBs: Multivesicular bodies
qRT-PCR: quantitative real-time polymerase chain reaction
rhLOXL4: recombinant human LOXL4
RT: Room temperature
SD: Standard deviation
SRCR: Scavenger receptor cysteine-rich
TBS: Tris-buffered saline
TCGA: The Cancer Genome Atlas
TNM: Tumor-node-metastasis
