# Supplementary material for: Exosome-mediated secretion of LOXL4 promotes hepatocellular carcinoma cell invasion and metastasis
Source: Mol Cancer. 2019 Jan 31;18:18. doi: 10.1186/s12943-019-0948-8 (PMC6354392; doi:10.1186/s12943-019-0948-8)
Supplement: Supplementary file 1 — Table S1. Primers for generation of LOXL4 deletion mutants. (DOCX 15 kb) [file 12943_2019_948_MOESM1_ESM.docx]

Additional Table S1 Primers for generation of LOXL4 deletion mutants

| Name | Sequence (5’-3’) |
| --- | --- |
| ΔSRCR | F: ctagctagcatggcgtggtccccaccagccaccctctttctgttcctgctgctgctaggccagccccctcccagcaggccaatggacagtgcaccagac  R: ttgcggccgctcagatgaggttgttcctgagacgctgttcctgctccagggagagttctgcattggctgggtatgaattccctgtgtggcagttgtgc |
| ΔC-T | F: ctagctagcatggcgtggtccccaccagc  R: ttgcggccgctcatgcactgtccatgcag |
| Full-length  (HA-tagged) | F: ctagctagcatggcgtggtccccaccagccaccctctttctgttcctgctgctgctaggccagccccct  R: ttgcggccgcagcgtaatctggaacatcgtatgggtaagaaccgccaccaccgatgaggttgttcctgag |
| ΔSRCR  (HA-tagged) | F: ctagctagcatggacagtgcaccagacctggtgatgaacgcccagctagtgcaggagacggcctacttgg  R: cgcggatccagcgtaatctggaacatcgtatgggtaagaaccgccaccaccgatgaggttgttcctgagac |
| ΔC-T  (HA-tagged) | F: ctagctagcatggcgtggtccccaccagccaccctctttctgttcctgctgctgctaggccagccccct  R: ttgcggccgcagcgtaatctggaacatcgtatgggtaagaaccgccaccacctgcactgtccatgcaggag |
