# Supplementary figures and images for: Exosome-mediated secretion of LOXL4 promotes hepatocellular carcinoma cell invasion and metastasis
Source: Mol Cancer. 2019 Jan 31;18:18. doi: 10.1186/s12943-019-0948-8 (PMC6354392; doi:10.1186/s12943-019-0948-8)

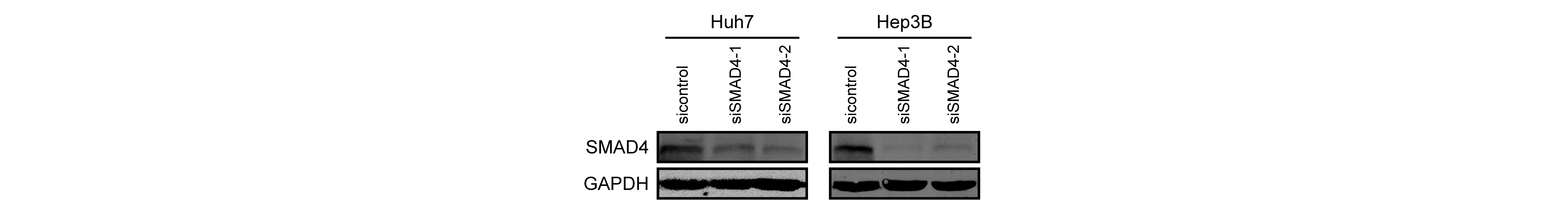

Supplement: Supplementary file 2 — Figure S1. The silencing efficacy of SMAD4 siRNAs in Huh7 and Hep3B cells was examined by western blotting. Figure S2. LOXL4 expression in HCC cell lines. a. LOXL4 expression at mRNA level in HCC cell lines examined by qRT-PCR. 18sRNA was detected as an internal control. b. LOXL4 expression at protein level in HCC cell lines examined by western blotting. Figure S3. Validation of LOXL4 overexpression and the effects of LOXL4 overexpression on cell proliferation and anoikis. a. QRT-PCR analysis of LOXL4 expression in LOXL4-overexpressing and control cells. b. Western blotting analysis of LOXL4 expression in LOXL4-overexpressing and control cells. c. Annexin V/PI stain by FACS was performed to measure anoikis rate after overexpression of LOXL4. d. Cell viability was measured by CCK-8 assay after overexpression of LOXL4. (** P < 0.01). Figure S4. Validation of LOXL4 knockdown and the effects of LOXL4 knockdown on cell proliferation. a. QRT-PCR analysis of LOXL4 expression in LOXL4 knockdown and control cells. b. Western blotting analysis of LOXL4 expression in LOXL4 knockdown and control cells. c. Cell viability was measured by CCK-8 assay after knockdown of LOXL4. Figure S5. The effects of LOXL4 knockdown on cell-matrix adhesion and the FAK/Src pathway are completely abolished by catalase. a. LOXL4 knockdown and control cells were subjected to cell-matrix adhesion assay to Col I, Col IV, LN, and FN with catalase treatment (500 U/ml) for 12 h. b. Cell migration potential was determined in LOXL4 knockdown and control cells upon treatment with vehicle or catalase according to Transwell assays. c. Western blotting analysis of phosphorylation of FAK and Src and total FAK and Src in LOXL4 knockdown and control cells with catalase treatment. (** P < 0.01). Figure S6. Examination of LOXL4 protein in the CM derived from LOXL4 knockdown cells and control cells by western blotting. Figure S7. a. Query results for gene symbol LOX in ExoCarta. b. The mRNA level of LOXL4 detected by [file 12943_2019_948_MOESM2_ESM.zip › Figure S1.tif]

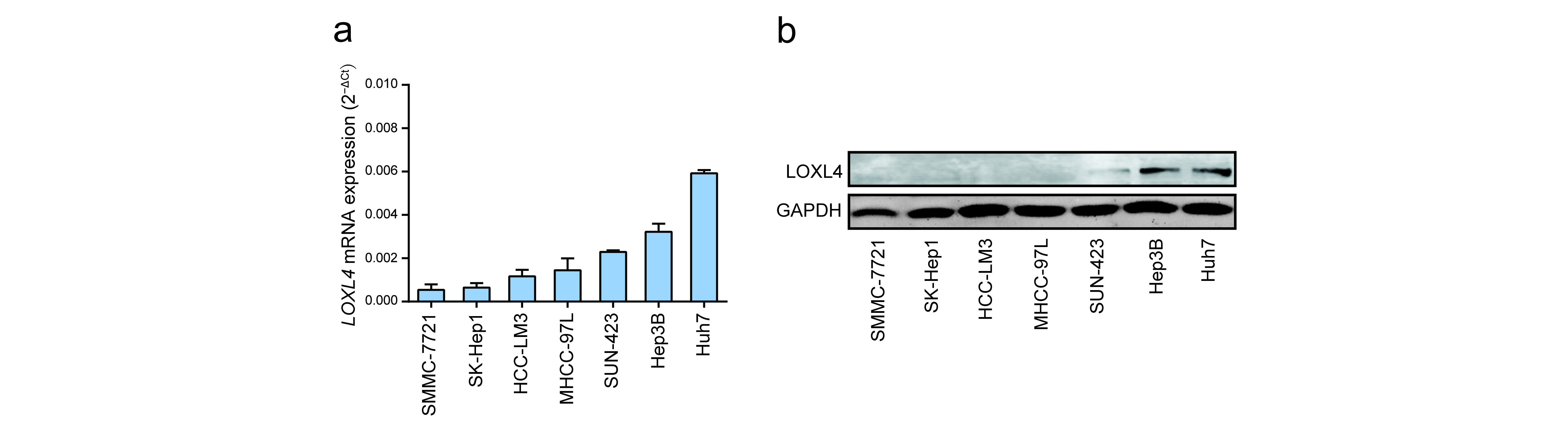

Supplement: Supplementary file 2 — Figure S1. The silencing efficacy of SMAD4 siRNAs in Huh7 and Hep3B cells was examined by western blotting. Figure S2. LOXL4 expression in HCC cell lines. a. LOXL4 expression at mRNA level in HCC cell lines examined by qRT-PCR. 18sRNA was detected as an internal control. b. LOXL4 expression at protein level in HCC cell lines examined by western blotting. Figure S3. Validation of LOXL4 overexpression and the effects of LOXL4 overexpression on cell proliferation and anoikis. a. QRT-PCR analysis of LOXL4 expression in LOXL4-overexpressing and control cells. b. Western blotting analysis of LOXL4 expression in LOXL4-overexpressing and control cells. c. Annexin V/PI stain by FACS was performed to measure anoikis rate after overexpression of LOXL4. d. Cell viability was measured by CCK-8 assay after overexpression of LOXL4. (** P < 0.01). Figure S4. Validation of LOXL4 knockdown and the effects of LOXL4 knockdown on cell proliferation. a. QRT-PCR analysis of LOXL4 expression in LOXL4 knockdown and control cells. b. Western blotting analysis of LOXL4 expression in LOXL4 knockdown and control cells. c. Cell viability was measured by CCK-8 assay after knockdown of LOXL4. Figure S5. The effects of LOXL4 knockdown on cell-matrix adhesion and the FAK/Src pathway are completely abolished by catalase. a. LOXL4 knockdown and control cells were subjected to cell-matrix adhesion assay to Col I, Col IV, LN, and FN with catalase treatment (500 U/ml) for 12 h. b. Cell migration potential was determined in LOXL4 knockdown and control cells upon treatment with vehicle or catalase according to Transwell assays. c. Western blotting analysis of phosphorylation of FAK and Src and total FAK and Src in LOXL4 knockdown and control cells with catalase treatment. (** P < 0.01). Figure S6. Examination of LOXL4 protein in the CM derived from LOXL4 knockdown cells and control cells by western blotting. Figure S7. a. Query results for gene symbol LOX in ExoCarta. b. The mRNA level of LOXL4 detected by [file 12943_2019_948_MOESM2_ESM.zip › Figure S2.tif]

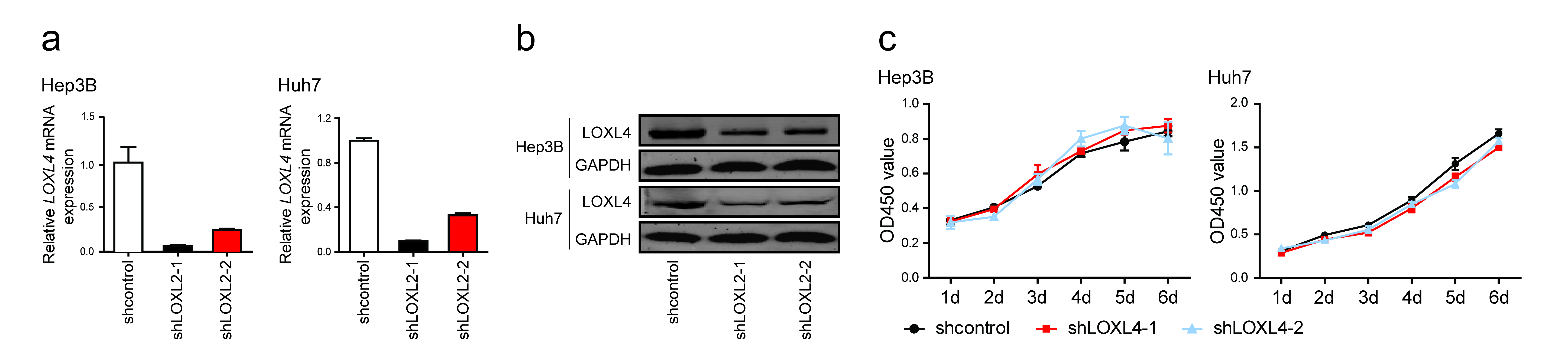

Supplement: Supplementary file 2 — Figure S1. The silencing efficacy of SMAD4 siRNAs in Huh7 and Hep3B cells was examined by western blotting. Figure S2. LOXL4 expression in HCC cell lines. a. LOXL4 expression at mRNA level in HCC cell lines examined by qRT-PCR. 18sRNA was detected as an internal control. b. LOXL4 expression at protein level in HCC cell lines examined by western blotting. Figure S3. Validation of LOXL4 overexpression and the effects of LOXL4 overexpression on cell proliferation and anoikis. a. QRT-PCR analysis of LOXL4 expression in LOXL4-overexpressing and control cells. b. Western blotting analysis of LOXL4 expression in LOXL4-overexpressing and control cells. c. Annexin V/PI stain by FACS was performed to measure anoikis rate after overexpression of LOXL4. d. Cell viability was measured by CCK-8 assay after overexpression of LOXL4. (** P < 0.01). Figure S4. Validation of LOXL4 knockdown and the effects of LOXL4 knockdown on cell proliferation. a. QRT-PCR analysis of LOXL4 expression in LOXL4 knockdown and control cells. b. Western blotting analysis of LOXL4 expression in LOXL4 knockdown and control cells. c. Cell viability was measured by CCK-8 assay after knockdown of LOXL4. Figure S5. The effects of LOXL4 knockdown on cell-matrix adhesion and the FAK/Src pathway are completely abolished by catalase. a. LOXL4 knockdown and control cells were subjected to cell-matrix adhesion assay to Col I, Col IV, LN, and FN with catalase treatment (500 U/ml) for 12 h. b. Cell migration potential was determined in LOXL4 knockdown and control cells upon treatment with vehicle or catalase according to Transwell assays. c. Western blotting analysis of phosphorylation of FAK and Src and total FAK and Src in LOXL4 knockdown and control cells with catalase treatment. (** P < 0.01). Figure S6. Examination of LOXL4 protein in the CM derived from LOXL4 knockdown cells and control cells by western blotting. Figure S7. a. Query results for gene symbol LOX in ExoCarta. b. The mRNA level of LOXL4 detected by [file 12943_2019_948_MOESM2_ESM.zip › Figure S4.tif]

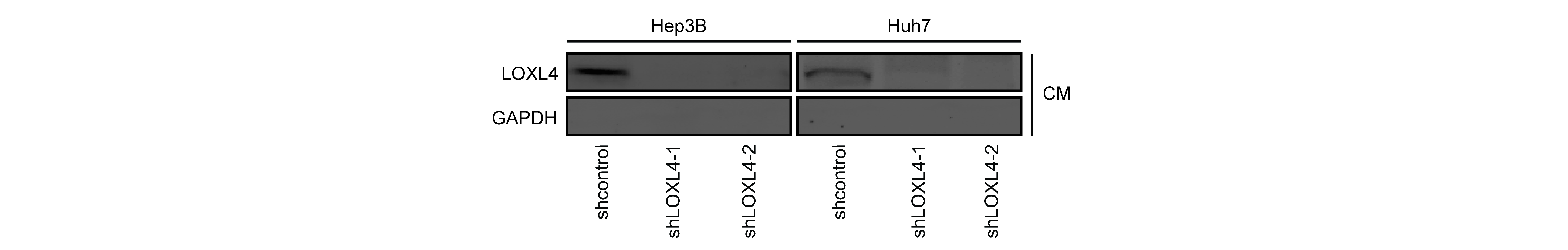

Supplement: Supplementary file 2 — Figure S1. The silencing efficacy of SMAD4 siRNAs in Huh7 and Hep3B cells was examined by western blotting. Figure S2. LOXL4 expression in HCC cell lines. a. LOXL4 expression at mRNA level in HCC cell lines examined by qRT-PCR. 18sRNA was detected as an internal control. b. LOXL4 expression at protein level in HCC cell lines examined by western blotting. Figure S3. Validation of LOXL4 overexpression and the effects of LOXL4 overexpression on cell proliferation and anoikis. a. QRT-PCR analysis of LOXL4 expression in LOXL4-overexpressing and control cells. b. Western blotting analysis of LOXL4 expression in LOXL4-overexpressing and control cells. c. Annexin V/PI stain by FACS was performed to measure anoikis rate after overexpression of LOXL4. d. Cell viability was measured by CCK-8 assay after overexpression of LOXL4. (** P < 0.01). Figure S4. Validation of LOXL4 knockdown and the effects of LOXL4 knockdown on cell proliferation. a. QRT-PCR analysis of LOXL4 expression in LOXL4 knockdown and control cells. b. Western blotting analysis of LOXL4 expression in LOXL4 knockdown and control cells. c. Cell viability was measured by CCK-8 assay after knockdown of LOXL4. Figure S5. The effects of LOXL4 knockdown on cell-matrix adhesion and the FAK/Src pathway are completely abolished by catalase. a. LOXL4 knockdown and control cells were subjected to cell-matrix adhesion assay to Col I, Col IV, LN, and FN with catalase treatment (500 U/ml) for 12 h. b. Cell migration potential was determined in LOXL4 knockdown and control cells upon treatment with vehicle or catalase according to Transwell assays. c. Western blotting analysis of phosphorylation of FAK and Src and total FAK and Src in LOXL4 knockdown and control cells with catalase treatment. (** P < 0.01). Figure S6. Examination of LOXL4 protein in the CM derived from LOXL4 knockdown cells and control cells by western blotting. Figure S7. a. Query results for gene symbol LOX in ExoCarta. b. The mRNA level of LOXL4 detected by [file 12943_2019_948_MOESM2_ESM.zip › Figure S6.tif]

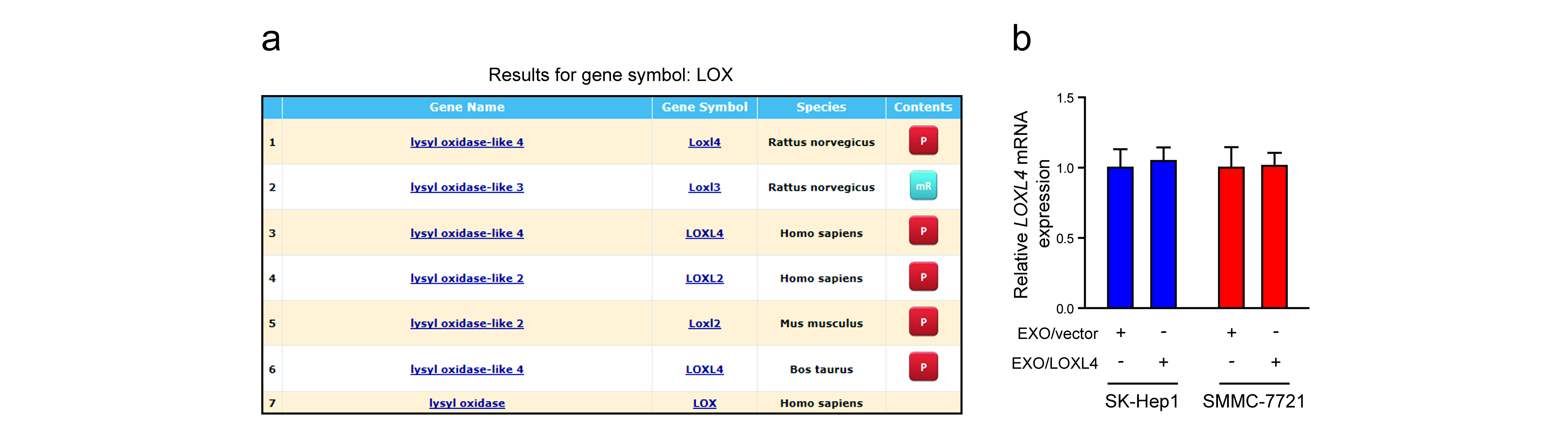

Supplement: Supplementary file 2 — Figure S1. The silencing efficacy of SMAD4 siRNAs in Huh7 and Hep3B cells was examined by western blotting. Figure S2. LOXL4 expression in HCC cell lines. a. LOXL4 expression at mRNA level in HCC cell lines examined by qRT-PCR. 18sRNA was detected as an internal control. b. LOXL4 expression at protein level in HCC cell lines examined by western blotting. Figure S3. Validation of LOXL4 overexpression and the effects of LOXL4 overexpression on cell proliferation and anoikis. a. QRT-PCR analysis of LOXL4 expression in LOXL4-overexpressing and control cells. b. Western blotting analysis of LOXL4 expression in LOXL4-overexpressing and control cells. c. Annexin V/PI stain by FACS was performed to measure anoikis rate after overexpression of LOXL4. d. Cell viability was measured by CCK-8 assay after overexpression of LOXL4. (** P < 0.01). Figure S4. Validation of LOXL4 knockdown and the effects of LOXL4 knockdown on cell proliferation. a. QRT-PCR analysis of LOXL4 expression in LOXL4 knockdown and control cells. b. Western blotting analysis of LOXL4 expression in LOXL4 knockdown and control cells. c. Cell viability was measured by CCK-8 assay after knockdown of LOXL4. Figure S5. The effects of LOXL4 knockdown on cell-matrix adhesion and the FAK/Src pathway are completely abolished by catalase. a. LOXL4 knockdown and control cells were subjected to cell-matrix adhesion assay to Col I, Col IV, LN, and FN with catalase treatment (500 U/ml) for 12 h. b. Cell migration potential was determined in LOXL4 knockdown and control cells upon treatment with vehicle or catalase according to Transwell assays. c. Western blotting analysis of phosphorylation of FAK and Src and total FAK and Src in LOXL4 knockdown and control cells with catalase treatment. (** P < 0.01). Figure S6. Examination of LOXL4 protein in the CM derived from LOXL4 knockdown cells and control cells by western blotting. Figure S7. a. Query results for gene symbol LOX in ExoCarta. b. The mRNA level of LOXL4 detected by [file 12943_2019_948_MOESM2_ESM.zip › Figure S7.tif]

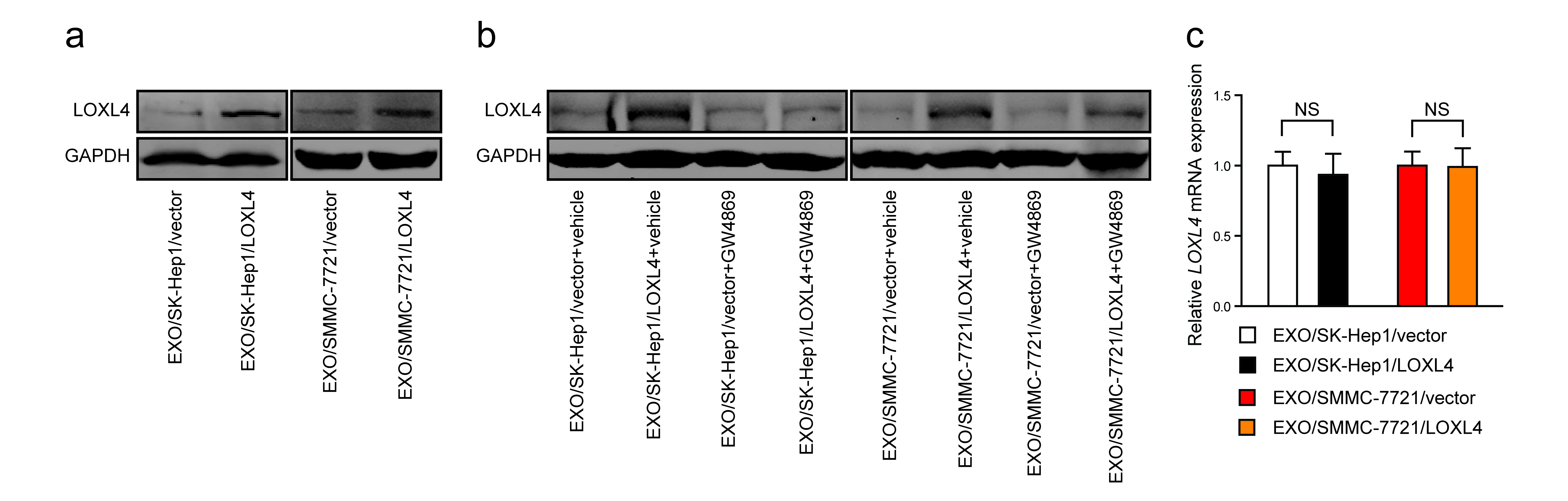

Supplement: Supplementary file 2 — Figure S1. The silencing efficacy of SMAD4 siRNAs in Huh7 and Hep3B cells was examined by western blotting. Figure S2. LOXL4 expression in HCC cell lines. a. LOXL4 expression at mRNA level in HCC cell lines examined by qRT-PCR. 18sRNA was detected as an internal control. b. LOXL4 expression at protein level in HCC cell lines examined by western blotting. Figure S3. Validation of LOXL4 overexpression and the effects of LOXL4 overexpression on cell proliferation and anoikis. a. QRT-PCR analysis of LOXL4 expression in LOXL4-overexpressing and control cells. b. Western blotting analysis of LOXL4 expression in LOXL4-overexpressing and control cells. c. Annexin V/PI stain by FACS was performed to measure anoikis rate after overexpression of LOXL4. d. Cell viability was measured by CCK-8 assay after overexpression of LOXL4. (** P < 0.01). Figure S4. Validation of LOXL4 knockdown and the effects of LOXL4 knockdown on cell proliferation. a. QRT-PCR analysis of LOXL4 expression in LOXL4 knockdown and control cells. b. Western blotting analysis of LOXL4 expression in LOXL4 knockdown and control cells. c. Cell viability was measured by CCK-8 assay after knockdown of LOXL4. Figure S5. The effects of LOXL4 knockdown on cell-matrix adhesion and the FAK/Src pathway are completely abolished by catalase. a. LOXL4 knockdown and control cells were subjected to cell-matrix adhesion assay to Col I, Col IV, LN, and FN with catalase treatment (500 U/ml) for 12 h. b. Cell migration potential was determined in LOXL4 knockdown and control cells upon treatment with vehicle or catalase according to Transwell assays. c. Western blotting analysis of phosphorylation of FAK and Src and total FAK and Src in LOXL4 knockdown and control cells with catalase treatment. (** P < 0.01). Figure S6. Examination of LOXL4 protein in the CM derived from LOXL4 knockdown cells and control cells by western blotting. Figure S7. a. Query results for gene symbol LOX in ExoCarta. b. The mRNA level of LOXL4 detected by [file 12943_2019_948_MOESM2_ESM.zip › Figure S8.tif]
